# Targeting a proteolytic neoepitope on CUB domain containing protein 1 (CDCP1) for RAS-driven cancers

**DOI:** 10.1172/JCI154604

**Published:** 2022-02-15

**Authors:** Shion A. Lim, Jie Zhou, Alexander J. Martinko, Yung-Hua Wang, Ekaterina V. Filippova, Veronica Steri, Donghui Wang, Soumya G. Remesh, Jia Liu, Byron Hann, Anthony A. Kossiakoff, Michael J. Evans, Kevin K. Leung, James A. Wells

**Affiliations:** 1 Department of Pharmaceutical Chemistry,; 2 Department of Radiology and Biomedical Imaging, and; 3 Helen Diller Family Comprehensive Cancer Center, UCSF, San Francisco, California, USA.; 4 Department of Biochemistry and Molecular Biology, and; 5 Institute for Biophysical Dynamics, The University of Chicago, Chicago, Illinois, USA.; 6 Preclinical Therapeutics Core, UCSF, San Francisco, California, USA.; 7 Chan Zuckerberg Biohub, San Francisco, California, USA.; 8 Department of Cellular and Molecular Pharmacology, UCSF, San Francisco, California, USA.

**Keywords:** Therapeutics, Antigen, Cancer immunotherapy, Proteases

## Abstract

Extracellular proteolysis is frequently dysregulated in disease and can generate proteoforms with unique neoepitopes not found in healthy tissue. Here, we demonstrate that Abs that selectively recognize a proteolytic neoepitope on CUB domain containing protein 1 (CDCP1) could enable more effective and safer treatments for solid tumors. CDCP1 is highly overexpressed in RAS-driven cancers, and its ectodomain is cleaved by extracellular proteases. Biochemical, biophysical, and structural characterization revealed that the 2 cleaved fragments of CDCP1 remain tightly associated with minimal proteolysis-induced conformational change. Using differential phage display, we generated recombinant Abs that are exquisitely selective to cleaved CDCP1 with no detectable binding to the uncleaved form. These Abs potently targeted cleaved CDCP1-expressing cancer cells as an Ab-drug conjugate, an Ab-radionuclide conjugate, and a bispecific T cell engager. In a syngeneic pancreatic tumor model, these cleaved-specific Abs showed tumor-specific localization and antitumor activity with superior safety profiles compared with a pan-CDCP1 approach. Targeting proteolytic neoepitopes could provide an orthogonal “AND” gate for improving the therapeutic index.

## Graphical abstract

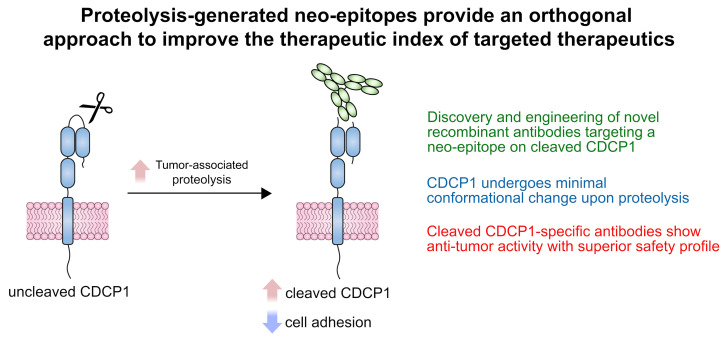

## Introduction

A key to safe and effective cancer treatments is to selectively target diseased over healthy cells. Traditionally, this has been enabled by targeting antigens with large expression differences between diseased and normal tissue, but this severely limits the druggable target space. More recently, there has been interest in targeting disease-associated alternate splice forms or peptide-MHC complexes (1, 2). These are often rare conformations, and their low abundance has posed challenges. Proteolysis plays critical roles in both normal and aberrant biological processes (3) and is well known to be upregulated and/or dysregulated in a variety of disease, including cancer (4, 5). Recently, there have been efforts to better understand disease-associated proteases and their substrates, and therapeutic strategies to inhibit proteases or utilize them for conditional activation of masked therapeutics have been explored (6–9). Tumor-associated proteolysis may also produce novel epitopes on the surface of cancer cells. We hypothesize that therapeutics that recognize these proteolytic neoepitopes could address the challenge of on-target, off-tumor toxicity. However, generation of Abs that can specifically recognize proteolytic neoepitopes (10), particularly those on the cell surface, has not been demonstrated.

Ras activation is one of the most well-known oncogenic transformations and is implicated in many solid tumors, including in nearly 90% of pancreatic cancer occurrences (11). CUB domain containing protein 1 (CDCP1), also known as Trask, gp140, SIMA135, and CD318, is a type I single-pass membrane protein that is highly overexpressed in a variety of solid tumors (12–14). CDCP1 is upregulated and functionally critical in KRAS-transformed cells, and overexpression of CDCP1 is associated with loss of adhesion, aggressive metastasis, and poor prognosis (15–17). Full-length CDCP1 (fl-CDCP1) is a 135 kDa protein composed of a large ectodomain, a transmembrane domain, and a short intracellular domain and is found on several types of tissue, including epithelial tissue along the gastrointestinal tract (18). The heavily glycosylated ectodomain of CDCP1 is predicted to contain 3 CUB-like domains, while the intracellular domain contains 5 tyrosine phosphorylation sites. Apart from this, the structure of CDCP1 has remained unknown.

Extracellular serine proteases have been shown to cleave CDCP1 into an N-terminal fragment (NTF) and a C-terminal fragment (CTF) (19). Proteolysis, along with overexpression, has been associated with the tumor-promoting functions of CDCP1, which involve phosphorylation of intracellular tyrosine residues and initiation of downstream signaling pathways associated with loss of adhesion, increased migration, and anoikis (20, 21). There is a higher proportion of cleaved CDCP1 (c-CDCP1) present in more aggressive metastatic cancer cell types compared with less malignant cancer cell lines (22). Given that protease levels and proteolytic activity are elevated in the tumor microenvironment (4), it is not surprising that c-CDCP1 is more prevalent in aggressive cancers (23, 24) and that normal tissues almost exclusively express fl-CDCP1 (25–27). Thus, we hypothesized that therapeutic agents that can specifically target c-CDCP1 over fl-CDCP1 may expand the therapeutic window.

Here, we report the biochemical and biophysical characterization of c-CDCP1 and the generation of recombinant Abs that specifically target this proteoform. We identified 3 cleavage sites of CDCP1 and found, surprisingly, that the NTF and CTF remained intact after proteolysis, with minimal structural rearrangement. The predicted structure of fl-CDCP1 by the recent AlphaFold (https://alphafold.ebi.ac.uk/) release corroborates our finding, revealing a large interface between NTF and CTF. Using a differential phage display strategy, we selected and optimized Abs that can specifically recognize an epitope on c-CDCP1 with no binding to uncleaved CDCP1. These Abs selectively target c-CDCP1–expressing pancreatic cancer cells and reduce tumor growth in vivo while substantially improving the safety profile compared with a pan-CDCP1–targeting approach. Ab-based targeting of proteolytic neoepitopes offers what we believe to be a novel strategy for expanding the therapeutic index.

## Results

### The NTF of CDCP1 is retained upon proteolysis.

**Figure 1:**
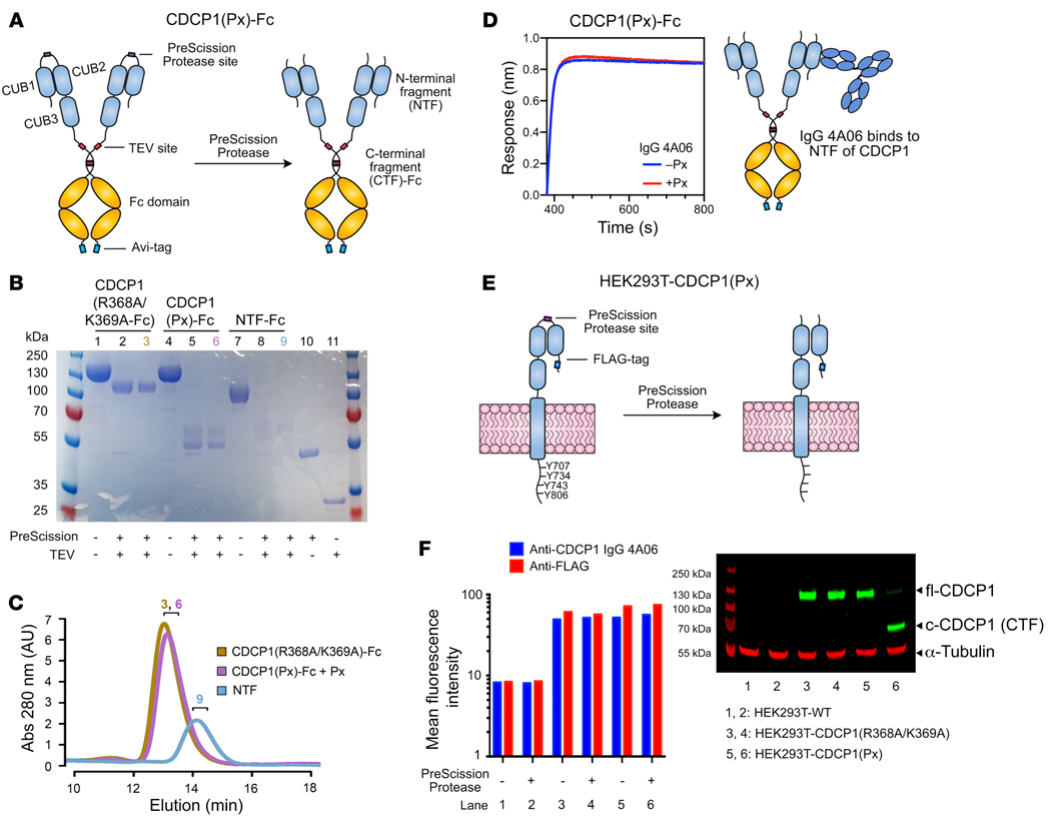
NTF of CDCP1 is retained upon proteolysis between the CUB1 and CUB2 domains. (**A**) Design of a Px-cleavable CDCP1 ectodomain fused to a TEV-releasable Fc domain with C-terminal avi-tag (CDCP1[Px]-Fc). The R368/K369 cleavage site was replaced with Px recognition sequence (GS)_5_-LEVLFQGP-(GS)_5_. (**B**) SDS-PAGE of CDCP1 constructs. Px treatment cleaves CDCP1(Px)-Fc into NTF and CTF-Fc fragments. NTF is heavily glycosylated (predicted 14 N-linked glycosylation sites) and runs as a smeared higher–molecular weight band at approximately 60 kDa. (**C**) SEC of CDCP1(R368/K369A)-Fc and CDCP1(Px)-Fc treated with Px and NTF (TEV released) shows that the NTF and CTF of CDCP1(Px) remain intact after proteolysis. Numbers denote fractions corresponding to the SDS-PAGE gel lanes in **B**. (**D**) BLI of IgG 4A06, which recognizes the NTF, shows robust binding to both Px-treated and untreated CDCP1(Px)-Fc. (**E**) Design of Px-cleavable CDCP1 with N-terminal FLAG-tag expressed in HEK293T cells. (**F**) Flow cytometry and Western blot of HEK293T-WT, HEK293T-CDCP1(R368A/K369A), and HEK293T-CDCP1(Px). Flow cytometry signal of anti-FLAG and IgG 4A06 remains unchanged with Px treatment. Western blot with anti-CDCP1 D1W9N, which recognizes the C-terminal intracellular region of CDCP1, shows Px-mediated CDCP1 proteolysis at the intended molecular weight.

CDCP1 has been reported to be cleaved at the dibasic residues between the NTF and CTF (R368/K369; ref. 18). To characterize CDCP1 proteolysis and target c-CDCP1, we first generated recombinant proteins and cell lines expressing CDCP1 with an engineered cut site between the NTF and CTF, where we replaced R368/K369 with a PreScission Protease (Px) recognition sequence to inducibly control CDCP1 proteolysis (CDCP1[Px]) (Figure 1A). We additionally generated a variant where R368 and K369 were mutated to alanine (CDCP1[R368A/K369A]-Fc) to prevent cleavage by basic residue–specific proteases and another where only the NTF (aa 30–369) was fused to an Fc domain (NTF-Fc). Efforts to recombinantly express the CTF (aa 370–665) alone were unsuccessful, suggesting NTF plays a role in expression and folding. Addition of Px cleaved CDCP1(Px)-Fc into 2 fragments (NTF and CTF) of expected molecular weight (Figure 1B). Surprisingly, we found that Px-treated CDCP1(Px)-Fc had the same size-exclusion chromatography (SEC) elution profile as uncleaved CDCP1-Fc, with no evidence of an unbound NTF (Figure 1C). Additionally, we observed robust binding by biolayer interferometry (BLI) of IgG 4A06, an Ab that recognizes the NTF of CDCP1 (Figure 1D and Supplemental Figure 1). To determine whether this was unique to Px cleavage, we generated and tested a thrombin protease–cleavable CDCP1-Fc (CDCP1[Tx]-Fc) (Supplemental Figure 2) and observed the same phenomena. To determine whether c-CDCP1 remains a complex on the cell membrane, we engineered cell lines expressing the fl-CDCP1 protein sequence with an N-terminal FLAG-tag and the R368/K369 proteolysis site replaced with the Px recognition sequence (Figure 1E). Using an anti-CDCP1 Ab (D1W9N) that recognizes the intracellular C-terminal region of CDCP1, we observed that, although addition of Px to cells cleaves CDCP1(Px) at the expected molecular weight, staining by anti-FLAG and IgG 4A06 was unaffected, indicating that the NTF remains membrane associated after proteolysis (Figure 1F).

### IP-MS reveals 3 major cleavage sites for CDCP1.

**Figure 2:**
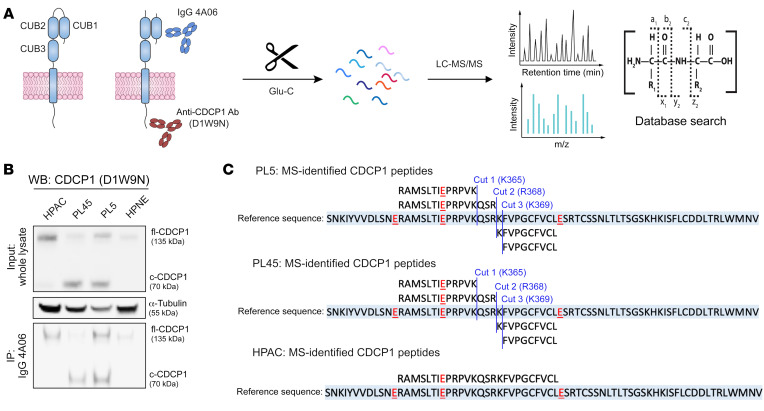
Identification of endogenous cut sites of CDCP1 on the surface of PDAC cells. (**A**) Schematic of IP-MS strategy to identify the endogenous proteolysis sites of CDCP1 on PDAC cells. CDCP1 was immunoprecipitated with IgG 4A06 or D1W9N Ab and digested with Glu-C, which cleaves after aspartic acid. Liquid chromatography/tandem mass spectrometry (LC-MS/MS) was used to identify peptides corresponding to proteolytic products of CDCP1. (**B**) Top: Western blot of PDAC cell lines expressing differential amounts of uncleaved CDCP1 and c-CDCP1. D1W9N Ab was used to detect CTF of CDCP1. PL5 and PL45 express mostly c-CDCP1, while HPAC expresses mostly uncleaved CDCP1. HPNE, a nonmalignant pancreatic cell line, expresses low levels of CDCP1. Bottom: IP blot shows that IP of NTF with IgG 4A06 can pull down the CTF of CDCP1. (**C**) CDCP1 peptides and proteolysis sites identified in PL5, PL45, and HPAC samples. Peptides identified by LC-MS for each cell line are aligned to the reference sequence (light blue). Glu-C cleavage sites are in red underlined text. Three proteolysis sites of CDCP1, cut 1 (K365), cut 2 (R368), and cut 3 (K369) are identified in PL5 and PL45 cells, but are absent in HPAC cells.

Confirming the precise proteolysis site(s) of CDCP1 on cancer cells is critical to designing the appropriate antigen for Ab generation. We used IP mass spectrometry (IP-MS) on a panel of pancreatic ductal adenocarcinoma (PDAC) cell lines expressing differential amounts of fl-CDCP1 and c-CDCP1 to map the proteolysis site(s) (Figure 2A). HPAC primarily expresses fl-CDCP1, whereas PL5 and PL45 express c-CDCP1, and HPNE is a nonmalignant pancreatic cell line with no CDCP1 expression (Figure 2B). Samples were treated with Glu-C to preserve the basic cut site(s) of c-CDCP1. We identified 3 unique cut sites after basic residues between the NTF and CTF; these included proteolysis after K365 (cut 1), R368 (cut 2), and K369 (cut 3). Cut 2 and cut 3 are previously reported proteolysis sites (28), while cut 1 appears to be a novel site (Figure 2C and Supplemental Figure 3). Peptides corresponding to the c-CDCP1 sequence were only observed in PL5 and PL45 cells, while only peptides mapping to the uncleaved sequence were found in HPAC and no CDCP1 peptides were observed for HPNE. These findings confirm that endogenous c-CDCP1 remains as a complex on PDAC cells and that CDCP1 is proteolytically processed between CUB1 and CUB2 to produce a heterogenous set of cleaved forms.

### Cleaved and uncleaved CDCP1 adopt similar conformations.

**Figure 3:**
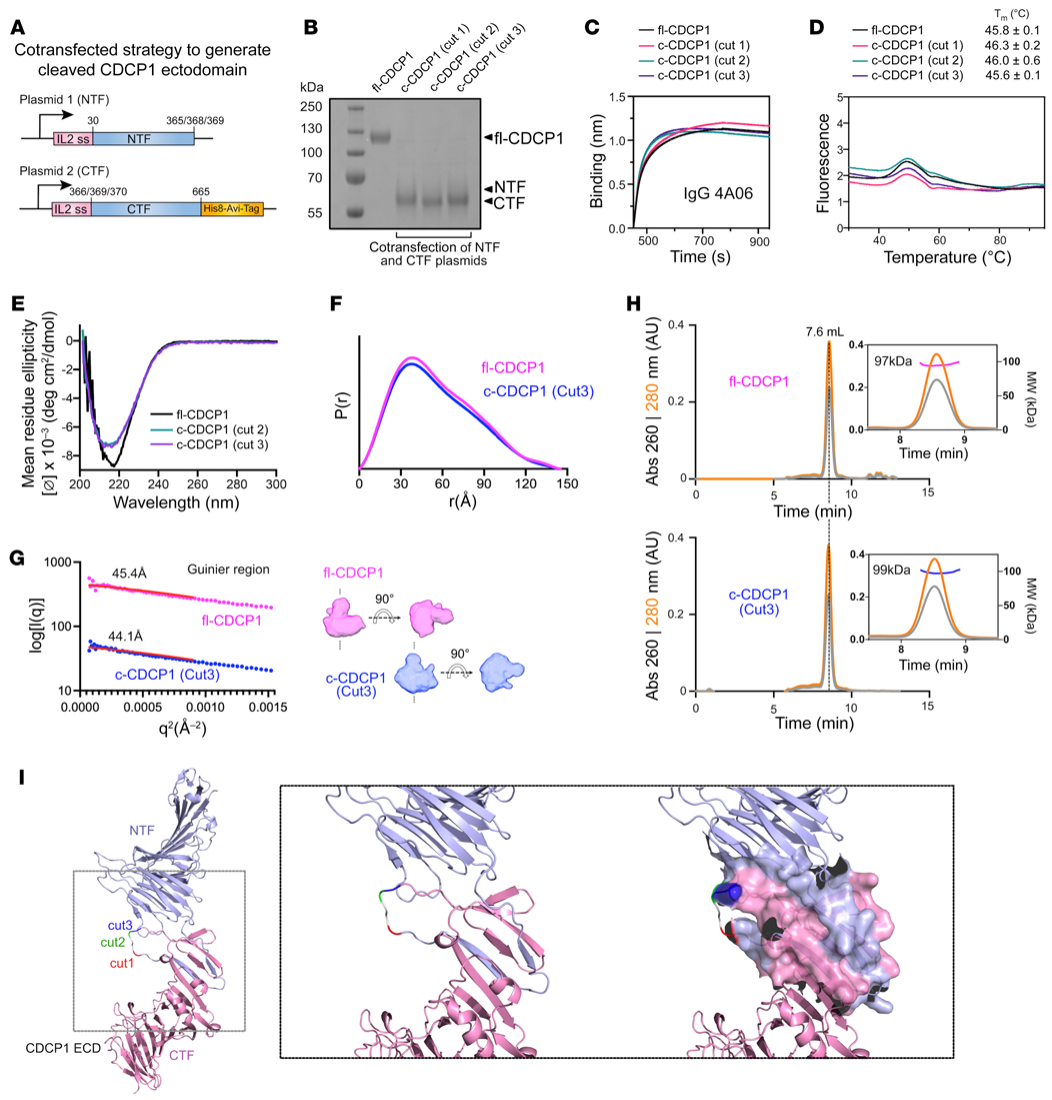
c-CDCP1 and uncleaved CDCP1 have similar conformations. (**A**) Schematic of the cotransfection strategy to generate c-CDCP1. The NTF and CTF are encoded on separate plasmids with an IL-2 signal sequence. (**B**) SDS-PAGE of fl-CDCP1 and c-CDCP1 (cut 1, cut 2, cut 3) ectodomain. NTF is heavily glycosylated and runs as a high–molecular weight smear. (**C**) BLI of IgG 4A06 to fl-CDCP1 or c-CDCP1 ectodomains shows that the NTF of CDCP1 is intact on c-CDCP1. (**D**) Differential scanning fluorimetry shows that fl-CDCP1 and c-CDCP1 have similar melting profiles and stabilities, suggesting that the NTF/CTF complex does not dissociate until unfolding of the full ectodomain. Melting temperature (*T_m_*) is reported as an average and SD of 2 replicates. (**E**) CD spectra of fl-CDCP1 and c-CDCP1. CDCP1 has a β sheet signature with minima of approximately 217 nm. The slight difference in spectral shape between fl-CDCP1 and c-CDCP1 indicates a subtle change in secondary structure. (**F**) SAXS-derived *P*(*r*) function of fl-CDCP1 and c-CDCP1 ectodomains. (**G**) *R_g_* and SAXS-derived ab initio envelopes of fl-CDCP1 and c-CDCP1 derived from SEC-SAXS show similar overall architecture. (**H**) SEC-MALS chromatograms of fl-CDCP1 and c-CDCP1 show similar elution profiles and molecular weights corresponding to monomeric ectodomain. (**I**) AlphaFold model of CDCP1 ectodomain. Residues involved in the NTF/CTF interface are shown as surface rendering in the inset.

We reasoned that because c-CDCP1 forms a tight NTF/CTF complex, we could generate recombinant c-CDCP1 with the native cut sites by cotransfection of the 2 fragments. Cotransfection of one set of plasmids encoding the NTF of c-CDCP1 with a second set of plasmids encoding the CTF ectodomain of c-CDCP1 resulted in an intact c-CDCP1 complex for all 3 cut variants (c-CDCP1 cut 1, cut 2, cut 3; Figure 3, A and B, and Supplemental Figure 4, A–D). fl-CDCP1 and c-CDCP1 both bound robustly to IgG 4A06 (Figure 3C) and had similar SEC profiles (Supplemental Figure 4E), demonstrating that c-CDCP1 forms a complex even when generated in trans. Remarkably, c-CDCP1 and fl-CDCP1 ectodomains have similar melting temperatures (Figure 3D), indicating that the NTF/CTF complex is stable and does not dissociate until unfolding of the entire ectodomain.

We examined the structure of fl-CDCP1 and c-CDCP1 ectodomains by circular dichroism (CD) spectroscopy (Figure 3E), SEC–small-angle X-ray scattering (SEC-SAXS) (Figure 3, F and G, and Supplemental Figure 5), and SEC–multi-angle light scattering (SEC-MALS) (Figure 3H). The CD spectra of fl-CDCP1 and c-CDCP1 indicated a classic β sheet signal, consistent with the CUB domain fold. There was a noticeable change in the spectral shape and minima between fl-CDCP1 and c-CDCP1, which suggests that proteolysis may cause subtle changes in the secondary structure of CDCP1. Comparison of the SEC-SAXS pair distance distribution functions and radii of gyration shows that both fl-CDCP1 and c-CDCP1 exhibit similar overall domain arrangement with no large-scale conformational changes as a result of proteolysis (Figure 3, F and G, and Supplemental Figure 5B). Furthermore, SEC-MALS showed that fl-CDCP1 and c-CDCP1 had the same elution profile and molecular weight (~97–99 kDa), consistent with the predicted size of a monomeric ectodomain (77 kDa plus glycosylation) (Figure 3H and Supplemental Table 1). Overall, these data show that, other than small differences in the β sheet signature, the conformation of fl-CDCP1 and c-CDCP1 are remarkably similar.

Recently, DeepMind released AlphaFold, which provides high-confidence structural predictions of virtually the entire human proteome (29). The AlphaFold prediction of the CDCP1 structure is remarkably consistent with our structural, biophysical, and biochemical data (Figure 3I). There is an extensive NTF/CTF interface with 2 β strands of the NTF interweaving with those of the CTF that is reinforced by multiple sidechain interactions. The loop containing the cleavage sites (cut 1, cut 2, cut 3) is solvent accessible and extends out of the NTF/CTF interface. The AlphaFold prediction further corroborates our experimental data and provides an atomistic model of the stable interaction between the NTF and CTF.

### Overexpression of both cleaved and uncleaved CDCP1 induces downstream signaling.

**Figure 4:**
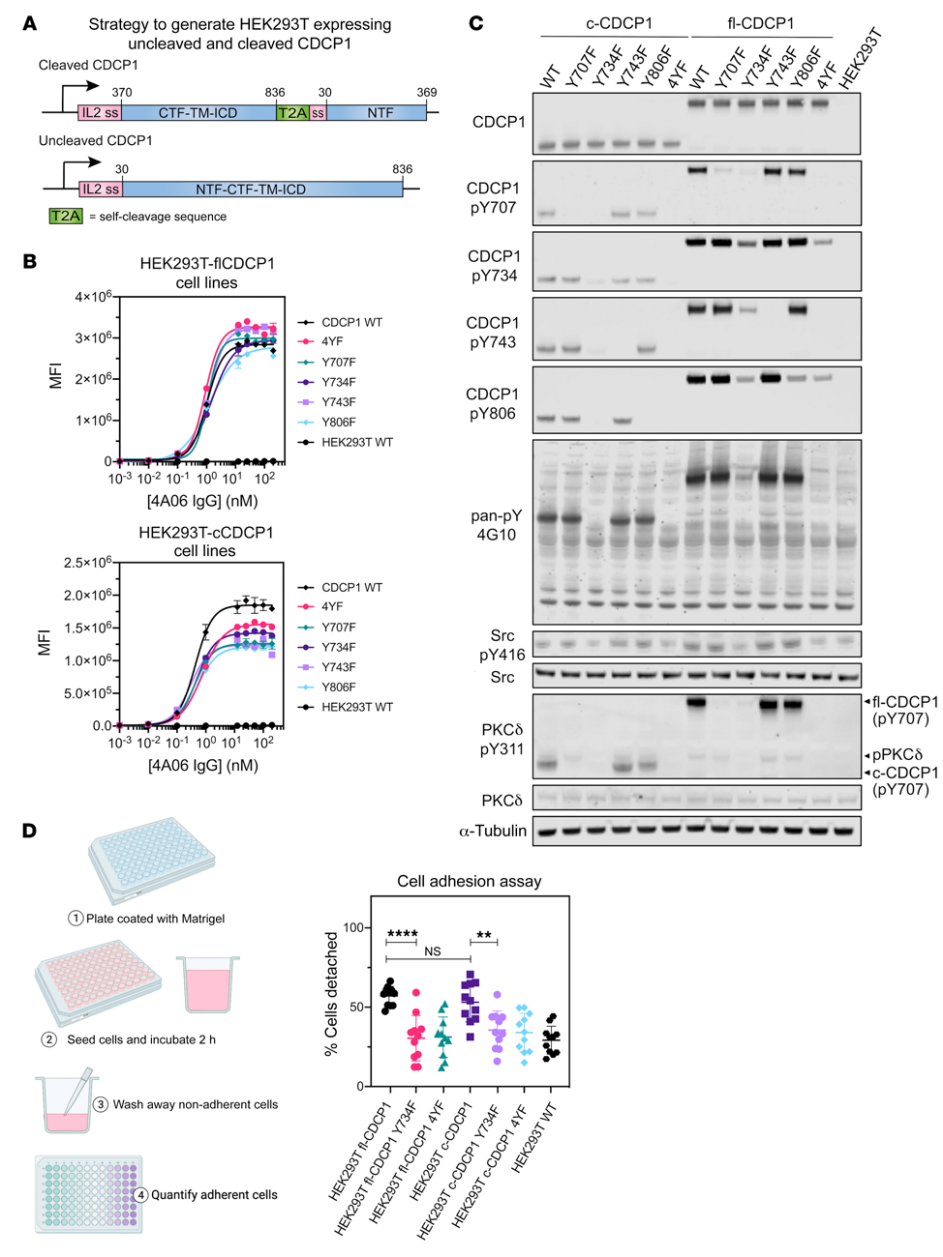
Both fl-CDCP1 and c-CDCP1 induce signaling and promote loss of adhesion. (**A**) Schematic of strategy to generate HEK293T cell lines expressing fl-CDCP1 or c-CDCP1. For c-CDCP1, a lentiviral vector was designed where a T2A self-cleavage sequence flanks the CTF (residues 370–836) and NTF (residues 30–369). For fl-CDCP1, a lentiviral vector encoding the full CDCP1 sequence (residues 30–836) was designed. An IL-2 signal sequence precedes each fragment. (**B**) Flow cytometry of IgG 4A06 to HEK293T fl-CDCP1 and HEK293T c-CDCP1 cell lines. Data are represented as mean ± SEM. *n* = 3. (**C**) Western blot of CDCP1 and intracellular proteins associated with CDCP1 signaling. Both fl-CDCP1 and c-CDCP1 are phosphorylated and initiate downstream signaling mediated by Src and PKCδ. Phosphorylation of Y734 on CDCP1 is important for phosphorylation of other tyrosine residues and Src and PKCδ. Anti-phosphoY311-PKCδ appears to be crossreactive to CDCP1-pY734. (**D**) Cell-adhesion assay shows that overexpression of both fl-CDCP1 and c-CDCP1 decreases cell adhesion and is dependent on phosphorylation of intracellular tyrosine residues, specifically of Y734. Data represent individual values and mean ± SEM. There was a significant difference in cell adhesion between the different cell lines (F[6, 70] = 10.98, *P* < 0.0001, 1-way ANOVA). Tukey’s post hoc tests revealed that overexpression of either fl-CDCP1 or c-CDCP1 decreased cell adhesion (*P* < 0.0001 for fl-CDCP1 vs. WT; *P* = 0.0001 for c-CDCP1 vs. WT), but adhesion of HEK293T fl-CDCP1 and HEK293T c-CDCP1 was not significantly different (NS, *P* = 0.98). The decreased cell adhesion was lost for both fl-CDCP1 and c-CDCP1 when all 4 intracellular tyrosine residues (4YF) or specifically Y734F was mutated (****P < 0.0001 for fl-CDCP1 vs. fl-CDCP1 Y734F and 4YF, **P = 0.011 for c-CDCP1 vs. c-CDCP1 Y734F, *P* = 0.004 for c-CDCP1 vs. c-CDCP1 4YF).

Overexpression of CDCP1 is associated with intracellular tyrosine phosphorylation and initiation of signaling pathways involving Src and PKCδ to promote protumorigenic processes, such as loss of adhesion and anoikis (30). To examine the function of the newly appreciated c-CDCP1 complex, we generated stable HEK293T cell lines expressing fl-CDCP1 or c-CDCP1 (Figure 4A). For fl-CDCP1, a lentiviral vector encoding the entire CDCP1 protein sequence was used. For c-CDCP1, we designed a vector in which a T2A self-cleaving sequence was placed between the CTF and the NTF. The T2A sequence was cleaved during translation to generate 2 polypeptides, enabling cell-surface expression of the c-CDCP1 complex from a single vector. We also generated variants where the 4 intracellular tyrosine residues were mutated to phenylalanine individually (Y707F, Y734F, Y743F, Y806F) or together (4YF; ref. 30). Flow cytometry (Figure 4B) and Western blot (Figure 4C; see complete unedited blots in the supplemental material) confirmed the successful generation of these stable cell lines. We found that both fl-CDCP1 and c-CDCP1 and downstream signaling partners Src and PKCδ were phosphorylated in these cell lines (Figure 4C). Additionally, Y734 is critical for the phosphorylation of the other intracellular tyrosine residues of fl-CDCP1 and c-CDCP1 and of Src and PKCδ (30). Overexpression of either fl-CDCP1 or c-CDCP1 decreased cell adhesion and was dependent on intracellular tyrosine phosphorylation, specifically of Y734 (Figure 4D and Supplemental Figure 6), while there was no significant effect on cell growth (Supplemental Figure 7). Because the expression levels of fl-CDCP1 and c-CDCP1 are not the same, we are not able to directly compare phenotypic differences between fl-CDCP1 and c-CDCP1 in this context. Regardless, these results collectively show that the c-CDCP1 NTF/CTF complex appears functional and reflect the known biology of CDCP1.

### IgG CL03 specifically recognizes the cleaved form of CDCP1.

**Figure 5:**
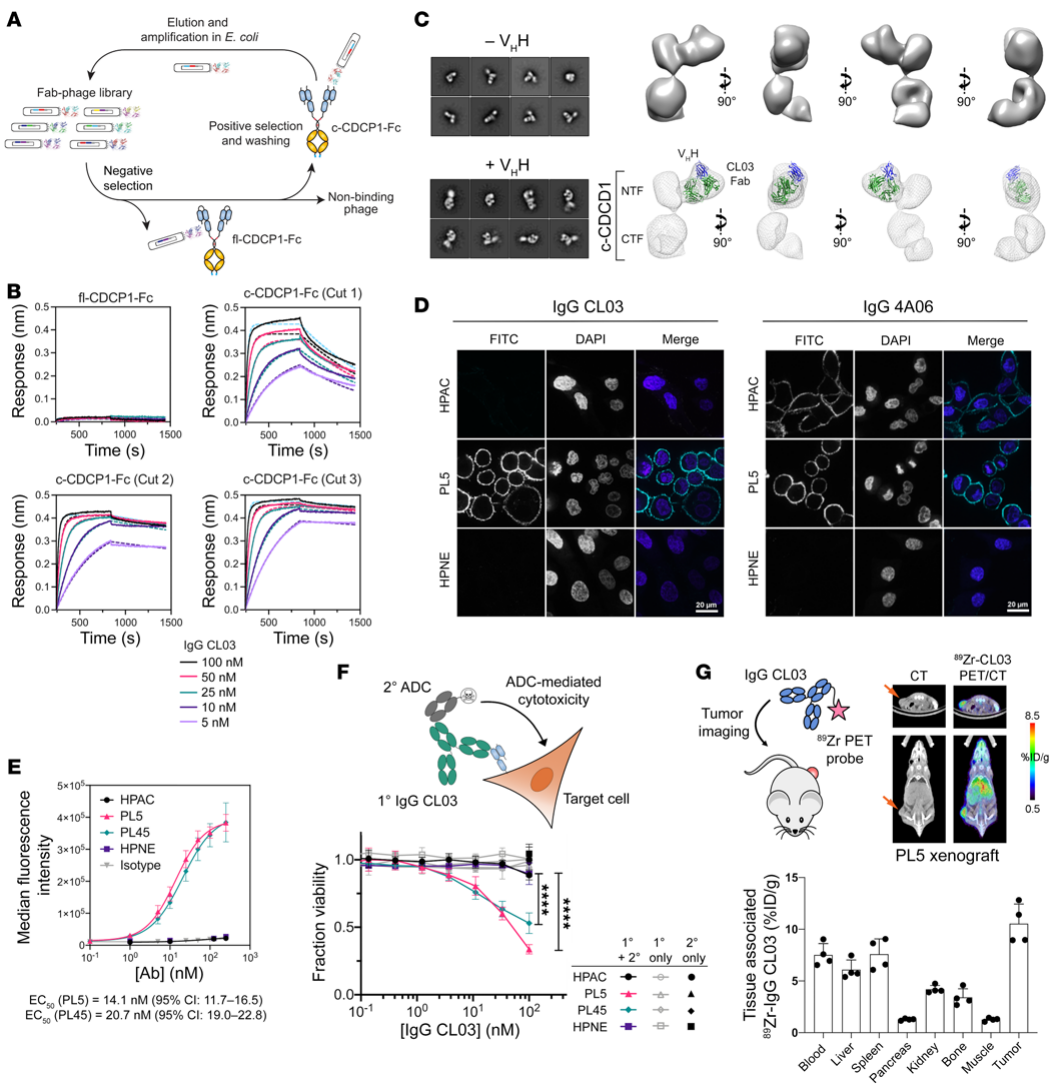
IgG CL03 specifically targets c-CDCP1–expressing pancreatic cancer cells. (**A**) Differential phage selection strategy to identify a c-CDCP1–specific Ab. Fab-phage was precleared with fl-CDCP1-Fc prior to positive selection with c-CDCP1-Fc. (**B**) BLI shows specific binding of IgG CL03 to c-CDCP1-Fc, but not to fl-CDCP1-Fc (*K_D_* = 150–840 pM, Supplemental Table 1). (**C**) Negative-stain EM 3D reconstruction of c-CDCP1 with CL03 Fab. Left: 2D class averages of c-CDCP1 (cut 3) plus CL03 Fab in the absence and presence of anti-Fab V_H_H. Right: 3D EM maps of CDCP1 (cut 3) plus CL03 Fab plus V_H_H with crystal structure of Fab (green) and V_H_H (blue) modeled into the density. (**D**) Immunofluorescence of HPAC, PL5, and HPNE cells with Alexa Fluor 488–labeled IgG CL03 (left panels) and IgG 4A06 (right panels). IgG CL03 specifically stains PL5 cells, while IgG 4A06 stains both HPAC and PL5 cells. No staining is observed for HPNE. Scale bars: 20 μm. (**E**) Flow cytometry shows that IgG CL03 binds to PL5 and PL45 cells, but not HPAC or HPNE cells. Data are represented as mean ± SEM. *n* = 3. (**F**) Top: schematic of ADC cell-killing assay. Bottom: dose-dependent ADC-mediated cell killing with IgG CL03 was only observed against PL5 and PL45 and only in the presence of both the primary and secondary Abs. Data are represented as mean ± SEM. *n* = 3. ****P < 0.0001, 2-way ANOVA. (**G**) In vivo PET imaging of ^89^Zr-labeled IgG CL03 in PL5 mouse xenografts shows tumor localization. Data represent individual values and mean ± SEM. *n* = 4.

To generate an Ab that can specifically recognize c-CDCP1, we employed a differential phage selection strategy using an in-house Fab-phage library (Figure 5A and ref. 31). Prior to each round of selection, the phage pool was cleared with fl-CDCP1 before positive selection with c-CDCP1. Purified antigens containing the 3 different cut sites were selected for individually or pooled. After 3 to 4 rounds of selection, there was enrichment for Fab-phage that bound c-CDCP1 over fl-CDCP1 (Supplemental Figure 8A). We identified a unique clone, CL03, that bound all 3 c-CDCP1 antigens selectively over fl-CDCP1 with subnanomolar IgG affinity (*K_D_* = 150–840 pM) (Figure 5B, Supplemental Figure 8B, and Supplemental Table 2). Plasmin is reported to be one of the proteases that can cleave CDCP1 (24, 32), and we found that IgG CL03 could also recognize plasmin-treated CDCP1 (Supplemental Figure 9, A–C).

We were interested in understanding how CL03 differentiates between fl-CDCP1 and c-CDCP1. It is possible CL03 could directly bind the cleavage “scars,” but it was challenging to rationalize how the Ab could recognize all 3 different cut sites with similar affinity. Alternatively, CL03 could bind an epitope that is unmasked upon proteolysis-induced conformational change. To investigate this, we tested the binding of CL03 to different CDCP1 constructs. We found that if the was immobilized via its C-termini, CL03 did not bind (Supplemental Figure 10, A and B). However, if the NTF was immobilized via its N-termini, CL03 bound NTF with affinity similar to that of c-CDCP1 (Supplemental Figure 10, B and C). This suggests that the CL03 epitope is located near the C-terminal portion of the NTF. Interestingly, we found that CL03 can also recognize an uncleaved CDCP1 variant where a 16 aa linker is inserted between NTF and CTF at the R368/K369 site (Supplemental Figure 10D). This indicates that, akin to proteolysis, extending the loop between the NTF and CTF can also unmask the CL03 epitope.

We obtained a 3D negative-stain electron microscopy (EM) reconstruction of c-CDCP1 ectodomain bound to CL03 Fab at 25 Å resolution and bound to 4A06 Fab at 23 Å resolution (Figure 5C and Supplemental Figure 11). A nanobody that binds at the “elbow” of the light chain was used as a fiducial mark to determine the orientation and “handedness” of the Fab. (33) c-CDCP1 adopted an elongated structure with 3 distinct “lobes” of density. We reasoned from our binding data that the Fab-bound domain was the NTF and that the other lobes belonged to the CTF. CL03 appeared to bind the NTF at a region proximal to the CTF, while 4A06 bound at the apex of the NTF at a distinct, nonoverlapping epitope. The AlphaFold model of CDCP1 docked well into the negative-stain EM maps of both fl-CDCP1 and c-CDCP1 obtained in the presence of Fab 4A06 (Supplemental Figure 11F). Interestingly, the AlphaFold model fit less well to the c-CDCP1 density when Fab CL03 was bound. It is possible that there are conformational rearrangements to c-CDCP1 that are induced by CL03 binding. Taking these data together, we propose a model in which the epitope of CL03 is located on the NTF proximal to the cut site, but is inaccessible in the uncleaved state. Proteolysis releases the C-termini of NTF to unmask this neoepitope and allow CL03 to bind (Supplemental Figure 10E). This could be achieved by rearrangement of the secondary structure elements of c-CDCP1, even while adopting an overall conformation similar to that of the uncleaved form.

### IgG CL03 targets c-CDCP1–expressing PDAC cells.

We then tested to determine whether our cleaved-specific Ab CL03 can specifically recognize c-CDCP1 on cancer cells. IgG CL03 stains c-CDCP1–expressing PL5 and PL45 cells with EC_50_ values of 14.1 and 20.7 nM, respectively, with no detectable binding to HPAC, which expresses fl-CDCP1, or HPNE, which does not express CDCP1 (Figure 5, D and E). Treating HPAC cells with plasmin increased binding of IgG CL03, suggesting that protease treatment can increase the amount of c-CDCP1 on the cell surface (Supplemental Figure 9, D and E). We then tested to determine whether an Ab-drug conjugate (ADC) strategy could be used to specifically deliver cytotoxic payloads to c-CDCP1–expressing PDAC cells. HPAC, PL5, PL45, and HPNE cells were treated with IgG CL03 as the primary Ab along with a secondary Ab conjugated to cytotoxin monomethyl auristatin F (MMAF) (Figure 5F). We observed dose-dependent cell killing of only PL5 and PL45 cells, while HPAC and HPNE were spared. Next, we tested to determine whether CL03, as a bispecific T cell engager (BiTE), could selectively recruit and activate immune cells in the presence of c-CDCP1–expressing target cells. Fab CL03 was genetically fused to an anti-CD3 OKT3 scFv and incubated with a Jurkat NFAT-GFP reporter cell line in coculture with PDAC cells (Supplemental Figure 12). We observed a dose-dependent increase in Jurkat NFAT activation in coculture with PL5 and PL45 cells, while coculture with HPAC and HPNE resulted in only baseline activation. Finally, we investigated the in vivo tumor localization of ^89^Zr-radiolabeled IgG CL03 in a PL5 mouse xenograft. PET imaging 48 hours after injection showed strong tumor localization of ^89^Zr-IgG CL03 (Figure 5G). Taken together, these studies demonstrate that IgG CL03 can selectively target c-CDCP1–expressing pancreatic cancer cells in a variety of modalities both in vitro and in vivo.

### IgG58, a cleaved-specific Ab to murine CDCP1, demonstrates antitumor activity with enhanced safety profile in a syngeneic mouse model.

**Figure 6:**
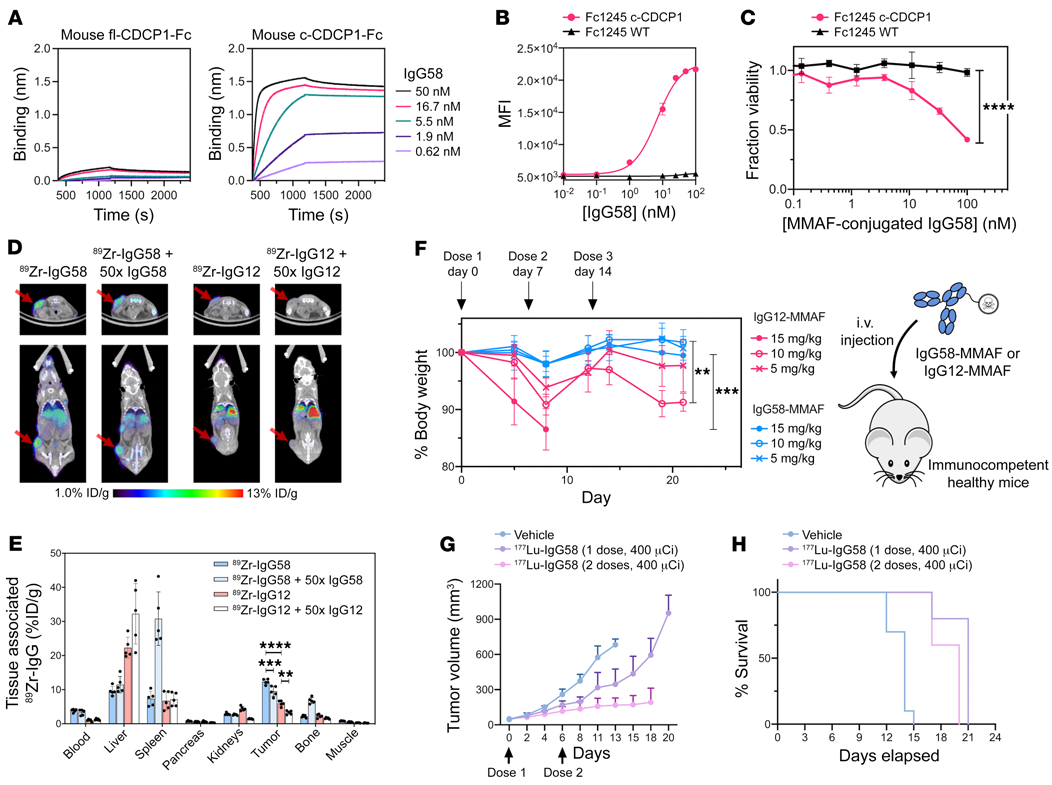
Efficacy of mouse c-CDCP1–specific Ab IgG58 in a syngeneic pancreatic tumor model. (**A**) BLI of IgG58 to mouse fl-CDCP1 and c-CDCP1. (**B**) Flow cytometry of IgG58. Data are represented as mean ± SEM. *n* = 3. (**C**) ADC-mediated cell killing of Fc1245 c-CDCP1 cells with IgG58-MMAF. Data are represented as mean ± SEM. *n* = 3. ****P = 0.0001, 2-way ANOVA. (**D**) Representative in vivo PET images and (**E**) biodistribution of ^89^Zr-IgG58 and ^89^Zr-IgG12 in mice harboring s.c. Fc1245 c-CDCP1 tumors (*n* = 5 per group). Data represent individual values and mean ± SEM. There was a significant difference in tumor signal across the treatment groups (F[3, 15] = 95.11, *P* < 0.0001, ANOVA). Tukey’s post hoc tests revealed both ^89^Zr-IgG58 and ^89^Zr-IgG12 signal decreased with administration of 50× unlabeled IgG, indicating target-specific localization. ***P = 0.0005 for IgG58; **P = 0.0013 for IgG12. There was significantly stronger tumor signal of ^89^Zr-IgG58 compared with ^89^Zr-IgG12, which shows weaker tumor localization and more widespread normal tissue distribution. ****P < 0.0001. (**F**) ADC toxicity assay in non–tumor-bearing mice. Mice (*n* = 5 per arm) were dosed weekly with 5, 10, or 15 mg/kg of IgG12-MMAF or IgG58-MMAF. There was a significant difference between the treatment arms (F[5,32] = 3.11, *P* = 0.0002, ANOVA). IgG58-MMAF treatment was better tolerated, with significant differences between IgG12-MMAF and IgG58-MMAF treatments at the 15 mg/kg dose (***P = 0.0068) and at the 10 mg/kg dose (**P = 0.0067) (Tukey’s multiple comparisons test). Data are represented as mean ± SEM. (**G** and **H**) Theranostic study of ^177^Lu-IgG58 (*n* = 5 mice per treatment arm, *n* = 8 for vehicle arm). Treatment with 400 μCi per dose of ^177^Lu-IgG58 resulted in decreased tumor growth and increased survival compared with vehicle. Data are represented as mean ± SEM. There was a statistically significant difference in tumor volume between vehicle group vs. ^177^Lu-IgG58 1-dose group (*P* = 0.0008) and vehicle group vs. ^177^Lu-IgG58 2-dose group (*P* < 0.0001), unpaired 2-tailed *t* test.

CL03 is crossreactive to cynomolgus, but not to mouse c-CDCP1 (Supplemental Figure 13). To enable syngeneic studies, we utilized the same differential phage display selection strategy to identify surrogate Abs specific to mouse c-CDCP1 (Supplemental Figure 14, A–C). After characterization and affinity maturation, we arrived at a lead mouse cleaved-specific CDCP1 Ab, IgG58, which binds mouse c-CDCP1 with high affinity and specificity (Figure 6A and Supplemental Table 3). We also identified IgG12, which, akin to the human CDCP1-specific IgG 4A06, recognizes both mouse fl-CDCP1 and c-CDCP1 with similar affinities (Supplemental Figure 14D). We also generated a stable mouse c-CDCP1 cell line in the background of Fc1245, an aggressive KPC model (Figure 4A and ref. 34). Both IgG58 and IgG12 recognize Fc1245 c-CDCP1 with an EC_50_ of 6.9 nM and 0.46 nM, respectively (Figure 6B and Supplemental Figure 14E). We further showed that IgG58 and IgG12, when reformatted to BiTE molecules, could activate Jurkat cells in the presence of Fc1245 c-CDCP1 cells (Supplemental Figure 14, F and G). Additionally, IgG58-MMAF and IgG12-MMAF ADC molecules could specifically deliver cytotoxic payloads to Fc1245 c-CDCP1 cells (Figure 6C and Supplemental Figure 14H).

We then tested the in vivo tumor localization of IgG12 and IgG58. ^89^Zr-IgG12 or ^89^Zr-IgG58 was injected into mice harboring subcutaneous Fc1245 c-CDCP1 tumors that were examined 48 hours later by PET imaging (Figure 6, D and E). High tumor localization of ^89^Zr-IgG58 was observed, and this signal decreased with coadministration of 50× unlabeled cold IgG58, indicating tumor-specific localization driven by specific target engagement. There was minimal ^89^Zr-IgG58 signal systemically. In contrast, we observed weaker tumor localization of ^89^Zr-IgG12 and more widespread off-tumor signal, indicating a higher presence of fl-CDCP1 in healthy tissues.

We proceeded to examine the safety profile of targeting cleaved versus uncleaved CDCP1. Non–tumor-bearing mice were dosed weekly with 5, 10, or 15 mg/kg of IgG12-MMAF or IgG58-MMAF, and their body weight was monitored for 21 days. None of the mice that received IgG58-MMAF at the 3 different doses exhibited significant changes in body weight (Figure 6F). In contrast, mice treated with IgG12-MMAF experienced significant body weight loss following each dose, indicative of treatment-induced toxicity. All mice receiving the 15 mg/kg dose of IgG12-MMAF had to be euthanized due to body weight loss by day 8, and 2 of the 5 mice receiving the 10 mg/kg dose of IgG12-MMAF were euthanized on day 19. These toxicity results suggest that a c-CDCP1 Ab would have a superior safety profile compared with a pan-CDCP1 targeting approach.

Finally, we investigated the antitumor activity of IgG58 as a radioligand therapeutic. Treatment of mice harboring subcutaneous Fc1245 c-CDCP1 tumors with 1 or 2 400 μCi doses of ^177^Lu-IgG58 resulted in significantly reduced tumor volume compared with vehicle control, with the 2-dose regimen approaching tumor stasis (Figure 6G and Supplemental Figure 15). Median survival for the treatment arms were 21 and 20 days for the 1- and 2-dose regimen, respectively, compared with 14 days for the vehicle group. The significant survival advantage imparted by ^177^Lu-IgG58 c-CDCP1 theranostic therapy (Figure 6H) supports our conclusion that Abs specific to proteolytic neoepitopes could expand the targetable disease space for cancer treatment. Compared with subcutaneous models, orthotopic mouse models are known to better simulate clinical prostate cancer, particularly with respect to the gene expression profiles and tumor microenvironment. In spite of little difference between these 2 models for extremely aggressive Fc1245 tumors, an orthotopic animal model is obviously the next step.

## Discussion

CDCP1 was first identified as a highly upregulated gene in colorectal and lung cancer (13) and has emerged as a driver of tumorigenesis and metastasis across a wide range of indications (12, 17, 20, 35). CDCP1 has been linked to a variety of oncogenic signaling networks, including Ras, EGFR, PDGFR, HER2, and HIF (15, 36, 37). Therapeutic interest in CDCP1 is reflected in numerous studies that report on small molecules (38) and Abs (16, 19, 32, 39–41) against CDCP1 and its pathways. However, despite these efforts, an anti-CDCP1 therapeutic has yet to enter the clinic. Although CDCP1 is highly expressed on cancer cells at close to approximately 2 million copies per cell (15, 16), it is also present on normal epithelial tissue (42). There is evidence that CDCP1 is not cleaved during normal physiological processes, but its cleavage is induced during tumorigenesis (43). This and other reports (25–27) suggest that c-CDCP1 would be rare on the surface of normal cells, and our work demonstrates that selectively targeting c-CDCP1 could be a safer, more therapeutically attractive approach. Continued work to characterize the prevalence and role of c-CDCP1, particularly in clinically relevant samples, will help determine which patient populations would be best suited to a c-CDCP1–targeting strategy.

Interestingly there is remarkably little conformational change between c-CDCP1 and fl-CDCP1. This presents a model for CDCP1 proteolysis and has been recently corroborated by Kryza et al. (44), who also observed that the NTF of CDCP1 does not dissociate upon proteolysis. By determining the exact sites of proteolysis and utilizing multiple biophysical and biochemical methods on both recombinant and cell lines, we further bolster the evidence that c-CDCP1 forms a complex and has a conformation similar to that of fl-CDCP1. Our structural, biophysical, and biochemical data are consistent with the structure predicted by AlphaFold and collectively provide what we believe is the most detailed structural examination of CDCP1 to date (29). The interweaving β sheets of the NTF and CTF form a highly stable β sheet bundle and an extensive interaction interface with the proteolysis site adjacent to this interface. We believe this model supports our findings that (a) upon proteolysis, the NTF stays tightly associated to the CTF, (b) there is little conformational change upon proteolysis, and (c) the CTF does not express in the absence of the NTF, due to the absence of the interweaved β sheet interactions to form a properly folded CTF. Furthermore, although CDCP1 has previously been predicted to have 3 CUB domains, the AlphaFold model shows that the NTF is composed of a single domain that does not adopt a CUB domain fold, while the CTF contains 2 CUB-like domains.

Extracellular neoepitopes generated by alternate splice forms, MHC-peptide complexes, posttranslational modifications (PTMs), and glycosylation are emerging as classes of therapeutic targets for cancer and other disease. However, characterizing these neoepitopes and developing therapeutic molecules that can selectively recognize them are challenging. Complications include, but are not limited to, low abundance of MHC-peptide complexes, rarity of alternative splice variants with targetable epitopes, glycoform heterogeneity, and identifying and validating truly disease-specific proteoforms. Proteolysis provides unique advantages in that it is irreversible, is highly prevalent in the tumor microenvironment, and can alter the conformation and structure of proteins and protein complexes in both subtle and marked ways. We show that proteolysis-generated neoepitopes on the cancer cell surface can provide an orthogonal approach to expanding the therapeutic index. The cleaved-specific CDCP1 Abs described here demonstrate that specifically targeting c-CDCP1 is effective and has a more favorable safety profile compared with targeting pan-CDCP1. Our work is a demonstration of an Ab specific to a proteolytically processed form of a cancer-associated cell-surface protein. Given the important and widespread role of proteases in disease biology, proteolysis-induced neoepitopes are likely widespread and could be targeted with Abs using a similar strategy. Recent unbiased proteomics methods that allow for detailed characterization of proteolysis on the cell surface will greatly increase the identification of disease-associated proteolytic neoepitopes (6).

Several serine proteases, such as plasmin (19, 28), matriptase (45), and uPA (28), have been shown to cleave CDCP1, are upregulated in solid tumors, such as pancreatic cancer (46), and are found in high levels in human clinical samples (47). Understanding the contributions of these proteases in the tumor microenvironment and any differences in activity and expression levels in patient subpopulations will inform our future strategy for proteolysis-targeted therapeutics. Solid tumors pose multiple therapeutic hurdles from a paucity of tumor-specific antigens, immunosuppressive microenvironment, hypoxia, and a complex stromal architecture (48). We anticipate that investigating these enhanced tumor-selective proteolysis markers, in combination with other traditional and emerging immunotherapy approaches (49), is likely to demonstrate the most efficacy and therapeutic benefit for patients.

## Methods

### Cloning, protein expression, and purification.

CDCP1, IgGs, and BiTEs were cloned into pFUSE. Fabs were subcloned into pBL347. pCDH-EF1-CymR-T2A-Neo was used for stable cell line cloning. Sequences were confirmed by Sanger sequencing. We used a previously described method for expression and purification of Fabs (31). CDCP1, IgGs, and BiTEs were generated by transfection of BirA-Expi293 cells using the ExpiFectamine 293 Transfection Kit (Life Technologies), purified by protein A or Ni-NTA affinity chromatography, and assessed by SDS-PAGE.

### Lentiviral cell line construction.

Stable cell lines were generated by lentiviral transduction. HEK293T Lenti-X cells were transfected with second-generation lentiviral packaging plasmids at approximately 80% confluence. FuGene HD (Promega) was used for transfection. After 72 hours, supernatant was harvested and filtered. Cleared supernatant was added to target cells with polybrene and centrifuged at 1000*g* at 33°C for 2 hours. Cells were incubated with viral supernatant overnight before the media was changed to fresh complete DMEM. Cells were expanded for 48 hours before being grown in drug-selection media. After 72 hours, cells were analyzed by flow cytometry for expression.

### Mammalian cell culture.

HPAC, PL5, PL45, and HPNE cells were a gift from the laboratory of E. Scott Seeley (Stanford University, Stanford, California, USA) and were maintained in IMDM plus 10% FBS plus 1× penicillin/streptomycin. HEK293T and Fc1245 cell lines were cultured in DMEM plus 10% FBS plus 1× penicillin/streptomycin. Jurkat NFAT-GFP cell lines were cultured in RPMI plus 10% FBS plus 2 mg/mL G418 plus 1× penicillin/streptomycin.

### IP.

Cells were washed with PBS and lysed with ice-cold NP-40 with protease inhibitor cocktail (Roche) and PhosSTOP (Roche). The lysate was incubated at 4°C for 30 minutes and centrifuged at 14,000*g* for 30 minutes at 4°C. For IP, CDCP1 Abs (D1W9N, Cell Signaling) were added to cell lysate precleared by protein A magnetic beads (EMD Millipore) and incubated overnight at 4°C. Abs were captured with protein A magnetic beads. For Western blot, 4× SDS loading buffer was added to beads and heated at 95°C for 5 minutes to elute protein. For IP-MS, protein was eluted with 0.1 M acetic acid and neutralized with pH 11 Tris.

### MS.

Samples were reduced with TCEP and alkylated with iodoacetamide. Proteins were digested with 20 μg sequencing-grade Glu-C (Promega) in 1 M urea at 37°C overnight. The samples were desalted using a Sola column (Thermo Fisher), dried, and dissolved in 0.1% formic acid plus 2% acetonitrile; 1 μg of peptide was injected into a prepacked 0.075 mm × 150 mm Acclaim Pepmap C18 LC column (2 μm pore size, Thermo Fisher) attached to a Q Exactive Plus (Thermo Fisher) mass spectrometer. Peptides were separated using a linear gradient of 3%–35% solvent B (solvent A: 0.1% formic acid; solvent B: 80% acetonitrile, 0.1% formic acid) over 170 minutes at 300 μL/min. MS1 and MS2 scans were collected in data-dependent acquisition mode using a top-20 method with a dynamic exclusion of 35 seconds and a charge exclusion restricted to charges of 2, 3, or 4. Peptide search and MS1 peak area quantification were performed using ProteinProspector (version 5.13.2).

### Western blot.

Immunoblotting was performed using the following antibodies: CDCP1 (D1W9N) (catalog 13794S), phospho-CDCP1 (Tyr707) (catalog 13111S), phospho-CDCP1 (Tyr806) (catalog 13024S), phospho-CDCP1 (Try734) (catalog 9050S), phospho-CDCP1 (Try743) (D2G2J) (catalog 14965S), Src (36D10) (catalog 2109S), phospho-Src family (Tyr416) (catalog 2101S), PKCδ (catalog 2058S), phospho-PKCδ (Tyr311) (catalog 2055S), and α-tubulin (DM1A) (catalog 3873S), imaged with LiCOR IRDye 680RD goat anti-mouse (catalog 925–68070), and IRDye 800CW goat anti-rabbit (catalog 926-32211) (all from Cell Signaling Technology).

### Flow cytometry.

Cells were lifted with Versene. Primary Abs were added for 30 minutes at 4°C and detected with the addition of Alexa Fluor 488 or Alexa Fluor 647–conjugated goat anti-human IgG, F(ab’)2 fragment specific (Jackson ImmunoResearch). Cells were analyzed using a CytoFLEX (Beckman Coulter) flow cytometer. All flow cytometry data analysis was performed using FlowJo software, version 10.8.1, and Prism software, version 9.3.1 (GraphPad).

### BLI.

BLI was performed using an Octet RED384 instrument (FortéBio). Biotinylated proteins were immobilized on a streptavidin (SA) biosensor, and His-tagged proteins were immobilized on a Ni-NTA biosensor. Affinities were calculated from a global fit (1:1) of the data using Octet RED384 data analysis software, version 12.0.

### SEC.

SEC was performed using an Agilent HPLC 1260 Infinity II LC System using an AdvanceBio SEC column (300 Å, 2.7 μm) in 0.15 M sodium phosphate. Each analyte was injected at 1 to 10 μM. Absorbance at 280 nm was monitored.

### CD spectroscopy.

CD spectra were measured using an Aviv 410 CD spectrophotometer. The CD signal from 200 nm to 300 nm was collected in a 0.1 cm path length cuvette at 25°C.

### SEC-SAXS and SEC-MALS.

SEC-SAXS data were collected at the SIBLYS beamline 12.3.1 of the Advanced Light Source at the Lawrence Berkeley National Laboratory (Berkeley, California, USA). Data were collected using a Dectris PILATUS3 2M detector at 20°C and processed as previously described (50). The SEC-SAXS flow cell was directly coupled with an Agilent 1260 Infinity HPLC System using a Shodex KW-803 column. The column was equilibrated with PBS pH 7.4 at 0.45 mL/min; 50 μL of protein was injected at approximately 5 mg/mL, and 3-second x-ray exposures were collected continuously. Radius of gyration (*R_g_*) was determined based on the Guinier approximation. Interference-free SAXS curves with least *R_g_* variation were averaged and merged in ScÅtter to produce the highest signal-to-noise SAXS curves. Pair distribution *P*(*r*) function was computed using program GNOM (51). *P*(*r*) functions were normalized based on molecular weight as determined based on their calculated constant volume (*V_c_*). MALS were collected using an 18-angle DAWN HELEOS II light scattering detector connected in tandem to an Optilab refractive index concentration detector (Wyatt Technology). MALS and differential refractive index data were analyzed using Wyatt Astra 7 software.

### Differential scanning fluorimetry.

2 μM protein in PBS was mixed with 4× Sypro Orange dye in a Bio-Rad 384-well PCR white plate and covered with qPCR Sealing Tape. The assay was performed over 25°C to 95°C with a temperature ramp rate of 0.5°C/30 s on a Roche LC480 Light Cycler.

### Negative-stain EM.

Complexes of c-CDCP1 with CL03 Fab and 4A06 Fab ± V_H_H were obtained by SEC on a Superdex 200 increase 10/300 GL column in 10 mM HEPES, 100 mM NaCl, pH 7.5; 5 μL of protein sample at 0.006–0.008 mg/mL was applied on a glow-discharged formvar/carbon-coated TEM grid and stained using a sequential 4-droplet method with 1% uranyl formate. Grids were screened on a FEI Tecnai G2 F30 300 kV Super Twin TEM Electron Microscope (FEI Company) at the Advanced Electron Microscopy Facility at the University of Chicago (Chicago, Illinois, USA). Micrographs were acquired at magnification 49 kX with a pixel size of 0.23 nm on the level of specimen using a 4K × 4K CCD camera. Particles were selected automatically using RELION (52). Extracted particles were 2D class averaged, sorted into initial classes, 3D classified, and refined in RELION. Final maps were analyzed in Chimera (53).

### Cell proliferation assay.

Cell proliferation assays were performed using a modified MTT assay to measure cell viability. 5000 Cells/well were plated in a 96-well plate on day 0 and incubated at 37°C under 5% CO_2_; 10 μL of 5 mg/mL of Thiazolyl Blue Tetrazolium Bromide (Sigma Aldrich) was added to each well and incubated at 37°C for 2 hours, and 100 μL of 10% SDS plus 0.01 M HCl was added to lyse the cells to dissolve the MTT product. After 4 hours, absorbance at 595 nm was measured using an Infinite M200 PRO-Plate Reader (Tecan).

### Cell adhesion assay.

On day 1, a 96-well tissue culture plate was coated with MaxGel ECM (MilliporeSigma) 1:10 diluted in serum-free DMEM. The culture medium for HEK293T cells was also changed to serum-free medium. The next day, media was removed and culture plates were blocked with 100 μL serum-free DMEM with 0.1 % BSA for 2 hours and washed with PBS; 100,000 cells in 100 μL of serum-free (0.1 % BSA) medium were added to each well and incubated at 37°C for 2 hours. The nonadherent cells were removed by washing with media 3 times, and the remaining cells were quantified by MTT assay.

### Phage selection and ELISA.

All phage selections were conducted according to previously established protocols (31). Briefly, selections were performed using biotinylated c-CDCP1-Fc captured on SA-coated magnetic beads (Promega). Prior to each selection, the phage pool was incubated with biotinylated fl-CDCP1-Fc captured on SA beads. Four rounds of selection were performed with decreasing amounts of antigen. Bound Fab-phage was eluted by the addition of TEV protease. Individual clones from the third and fourth round of selection were analyzed by phage ELISA. Phage ELISA was performed according to standard protocols and detected using HRP-conjugated anti-M13 phage Abs (GE Lifesciences, 27-9421-01) using a TMB substrate.

### Immunofluorescence.

HPAC, PL5, and HPNE cells were plated on glass-bottom imaging plates (MatTek) and incubated for 24 hours at 37°C. Cells were treated with IgG (1 μg/mL) for 30 minutes and washed with media to remove unbound IgG. Bound IgG was detected by the addition of Alexa Fluor 488–conjugated AffiniPure F(ab′)2 fragment goat anti-human IgG, F(ab′)2 fragment specific (Jackson ImmunoResearch, 143225), in Invitrogen Molecular Probes Live Cell Imaging Solution (Thermo Fisher) containing Hoescht blue (2 μg/mL). Cells were imaged on a Nikon Ti Microscope Yokogawa CSU-22 with spinning disk confocal.

### In vitro ADC assays.

Secondary ADC assays used a Fab anti-human IgG Fc-MMAF Ab with cleavable linker (Moradec). For ADC assays using direct conjugation, the Ab was labeled with DBCO-PEG4-ValCit-MMAF (Levena Biosciences) at residue T74M of the light chain using oxazirdine chemistry following a previously described protocol (54). 5000 Cells/well were seeded on a 96-well polylysine-coated white plate. The next day, media was removed and primary IgG and secondary ADC at a 1:4 ratio or MMAF-labeled IgG was added. Cells were incubated for 72 hours, and viability was measured using CellTiter-Glo Reagent (Promega).

### BiTE assay.

25,000 Target cells/well were seeded on a 96-well plate. The next day, 50,000 Jurkat NFAT-GFP reporter cells/well and BiTE were added. After 20 hours, Jurkat cells were recovered by gentle pipetting and washed in PBS plus 3% BSA; GFP expression was quantified by flow cytometry using a CytoFLEX (Beckman Coulter) flow cytometer.

### ^89^Zr-IgG PET/CT mouse imaging studies.

1 mg of IgG was mixed with 0.1 M sodium bicarbonate pH 9.0. p-Isothiocyanatobenzyl-desferrioxamine (Df-Bz-NCS) was added at 3-fold molar excess. After 60 minutes at 37°C, the reaction mixture was purified into PBS pH 7.4 via a G-25 column. In a reaction vial, ^89^Zr-oxalic acid solution (5 mCi; 10 μL) was neutralized with 200 μL of 1 M HEPES pH 7.4, and 0.5 mg of IgG-DFO was added to the reaction vial. After 60 minutes at 37°C, radiolabeling efficiency was determined by instant thin layer chromatography (ITLC) using 20 mM citric acid (pH 4.9–5.1). The radiolabeling efficiency was consistently greater than 98.5%. Purification and buffer exchange into PBS were performed using a G-25 column. ^89^Zr-IgG was further diluted with 0.9% sodium chloride injection, USP, before i.v. administration for PET imaging.

Four- to 6-week-old intact male athymic nu/nu mice (Charles River Laboratory) bearing subcutaneous PL5 tumors were used for IgG CL03 PET imaging. Six- to eight-week-old healthy immunocompetent male black 6 (C57BL/6J) mice (Jackson Laboratory) bearing subcutaneous Fc1245 c-CDCP1 tumors were used for IgG58 or IgG12 PET imaging. Tumor-bearing mice received 200 μCi of labeled Abs in 100 μL saline using a custom mouse tail-vein catheter. After 48 hours, mice were anesthetized with isoflurane and imaged on a small-animal PET/CT scanner (Inveon, Siemens Healthcare). Decay corrected images were analyzed using AMIDE software, version 1.0.5. Forty-eight hours after radiotracer injection, animals were euthanized by cervical dislocation. Blood was harvested via cardiac puncture. Tissues were removed, weighed, and counted on a Hidex Automatic Gamma Counter for accumulation activity. The mass of the injected radiotracer was measured and used to determine the total cpm by comparison with a standard of known activity. The data were background and decay corrected and presented as the percentage of the injected dose/weight of the biospecimen in grams (%ID/g).

### Mouse ADC toxicity study.

The ADC labeling protocol was adapted from previous reports (55). The ADC toxicity study was performed with 8- to 10-week-old healthy male black 6(C57BL/6J) mice (Jackson Laboratory). Mice (*n* = 5 per group) were dosed i.v. weekly for 3 weeks with ADC (15, 10, 5 mg/kg in 200 μL PBS). Body weight was monitored biweekly for 4 weeks. Mice were euthanized if body weight dropped below 80%.

### ^177^Lu-IgG mouse study.

2 mg of IgG58 was mixed with 0.1 M sodium bicarbonate pH 9.0. S-2-(4-isothiocyanatobenzyl)-1,4,7,10-tetraazacyclododecane tetraacetic acid (p-SCN-Bn-DOTA) was added at 50-fold molar excess. After 90 minutes at 37°C, the IgG58-DOTA was purified via a G-25 column preequilibrated with 0.2 M ammonium acetate pH 7.0. In a reaction vial, ^177^Lu-chloride solution (6 mCi; 3 μL) was added directly to IgG58-DOTA. After incubation at 37°C for 60 minutes, radiolabeling efficiency was determined by ITLC. Radiolabeling efficiency was consistently greater than 98.5%. Purification and buffer exchange into PBS was performed with a PD-10 column. The purified ^177^Lu-IgG58 was further diluted with 0.9% sodium chloride injection, USP, before being administered into mice.

Four- to six-week-old healthy male black 6 (C57BL/6J) mice (Jackson Laboratory) were inoculated s.c. with Fc1245 c-CDCP1 (~1.0 × 10^6^ cells) in a 1:1 mixture (v/v) of media (DMEM) and Matrigel (Corning). Treatments were started 3 days after tumor implantation. Mice received ^177^Lu-IgG58 or vehicle (saline) at the indicated dose via tail-vein injection. Mice were weighed and tumor size measured 3 times a week until the completion of the study. End points were euthanasia due to tumor volume of greater than 2000 mm^3^, tumor ulceration, or greater than 20% loss in body weight.

### Statistics.

All graphing and statistical analysis was performed in GraphPad Prism (version 8.4.2). For cell adhesion assay, unpaired, 2-tailed *t* test was used. For the ADC toxicity study, statistical analysis was performed using 1-way ANOVA. For the mouse efficacy study, unpaired, 2-tailed *t* test was used. *P* values of less than 0.05 were considered statistically significant.

### Study approval.

All mouse studies were conducted in compliance with and were approved by the IACUC at UCSF.

## Author contributions

SAL and JZ designed and conducted all experiments unless otherwise noted. AJM conducted the characterization of PreScission-cleavable CDCP1. YHW and MJE conducted or supervised the PET imaging experiments. EVF and AAK conducted or supervised EM data collection and analysis. VS, DW, and BH conducted or supervised the mouse experiments. SGR prepared and analyzed the SAXS experiments. JL conducted the differential scanning fluorimetry experiments. KKL and JAW supervised the research. SAL, JZ, and JAW prepared and wrote the manuscript with input from all authors. SAL and JZ are co–first authors and contributed equally to the study. Their authorship order was chosen as alphabetical order by last name.

## Supplementary Material

Supplemental data

## Version 1 02/15/2022

Electronic publication
