# Supplementary material for: Targeting a proteolytic neoepitope on CUB domain containing protein 1 (CDCP1) for RAS-driven cancers
Source: J Clin Invest. 2022 Feb 15;132(4):e154604. doi: 10.1172/JCI154604 (PMC8843743; doi:10.1172/JCI154604)

Supplemental material for:

Targeting a proteolytic neo-epitope on CUB-domain containing protein 1 (CDCP1) for RAS-driven cancers

Authors: Shion A. Lim[1]*‡, Jie Zhou[1]*, Alexander J. Martinko[1]§, Yung-Hua Wang[2,3], Ekaterina V. Filippova[4,5], Veronica Steri[3,6], Donghui Wang[3,6], Soumya G. Remesh[1], Jia Liu[1]¶, Byron Hann[6], Anthony A. Kossiakoff[4,5], Michael J. Evans[2,3], Kevin K. Leung[1], James A. Wells[1,7,8,#]

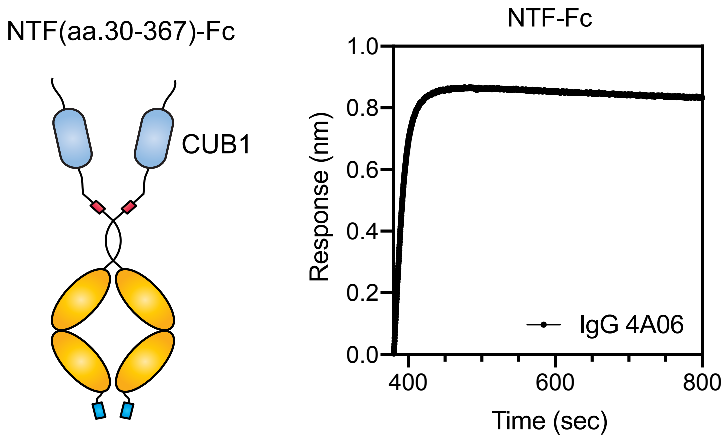

Supplementary Figure 1: BLI of IgG 4A06 binding to the N-terminal fragment (NTF) of CDCP1. NTF, composed of residues 30-367 of CDCP1, fused to a C-terminally biotinylated Fc domain was immobilized on Streptavidin-coated biosensors and probed with 25 nM of IgG 4A06. IgG 4A06 binds robustly to NTF-Fc, indicating that 4A06 recognizes an epitope within the NTF of the CDCP1 ectodomain.

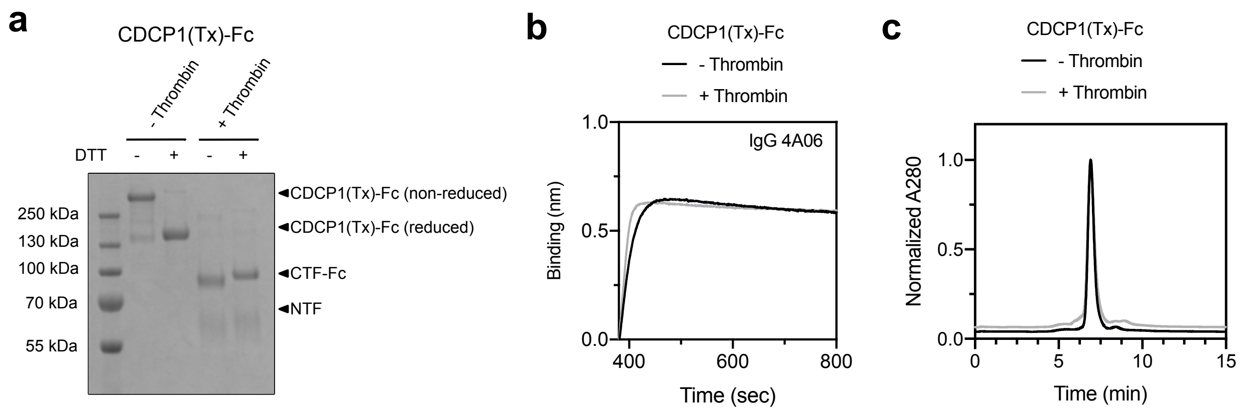

Supplementary Figure 2: Thrombin treatment of CDCP1 ectodomain with engineered thrombin cut site generates cleaved CDCP1 complex. (**a**) SDS-PAGE gel of Thrombin protease-cleavable CDCP1-Fc (CDCP1(Tx)-Fc). The reported R368/K369 cleavage site was replaced with a Thrombin protease recognition sequence $(GS)_5$-LVPRGS-$(GS)_5$. Treatment with Thrombin protease cleaves CDCP1(Tx)-Fc between the CUB1 and CUB2 domains and generate the corresponding NTF and CTF-Fc fragments. (**b**) BLI of IgG 4A06 binding to C-terminally immobilized CDCP1(Tx)-Fc treated or untreated with Thrombin shows that IgG 4A06, that recognizes the NTF, can still bind cleaved CDCP1. (**c**) SEC of CDCP1(Tx)-Fc treated or untreated with Thrombin protease shows that cleaved and uncleaved CDCP1 have similar elution profiles suggesting the NTF and CTF-Fc remain associated.

a **PL5 CDCP1 Peptide Coverage**

1 MAGLNCGVSI ALLGVLLLGA ARLPRGAEA**F EIALPRE**SNI TVLIKLGTPT LLAKPCYIVI SKRHITMLSI KSGERIVFTF
81 SCQSPENHFV IE**IQKNIDCM SGPCPFGE**VQ LQPSTSLLPT LNRTFIWDVK AHKSIGLELQ FSIPRLRQIG PGESCPDGVT
161 HSISGRIDAT VVRIGTFCSN GTVSRIKMQE GVKMALHLPW FHPRNVSGFS IANRSSIKRL CIIESVFEGE GSATLMSANY
241 PEGFPEDELM TWQFVVPAHL RASVSFLNFN LSNCERKEE**R VEYYIPGSTT NPE**VFKLEDK QPGNMAGNFN LSLQGCDQDA
321 QSPGILRLQF QVLVQHPQNE **SNKIYVVDLS NERAMSLTIE PRPVKQSRKF VPGCFVCLE**S RTCSSNLTLT SGSKHKISFL
401 CDDLTRLWMN VEKTISCTDH RYCQRKSYSL QVPSDILHLP VELHDFSWKL LVPKDRLSLV LVPAQKLQQH THEKPCNTSF
481 SYLVASAIPS QDLYFGSFCP GGSIKQIQVK QNISVTLRTF APSFQQE**ASR QGLTVSFIPY FKEE**GVFTVT PDTKSKVYLR
561 TPNWDRGLPS LTSVSWNISV PRDQVACLTF FKE**RSGVVCQ TGRAFMIIQE** QRTRAEEIFS LDEDVLPKPS FHHHSFWVNI
641 SNCSPTSGKQ LDLLFSVTLT PRTVDLTVIL IAAVGGGVLL LSALGLIICC VKKKKKKTNK GPAVGIYNDN INTEMPRQPK
721 KFQKGRKDND SHVYAVIE**DT MVYGHLLQDS SGSFLQPEVD TYRPFQG**TMG VCPPSPPTIC SRAPTAKLAT EEPPPRSPPE
801 SESEPYTFSH PNNGDVSSKD TDIPLLNTQE PMEPAE

m/z	z	ppm	DB Peptide	Constant Mods	Variable Mods	RT	Score	Expect
926.9037	2	-1.4	IQKNIDCMSGPCPFGE	Carbamidomethyl@7; Carbamidomethyl@12		73.341615	32.6	8.9e-13
499.9567	3	-0.92	RAMSLTIEPRPVK			45.8198360	26.9	1.6e-11
468.0182	4	1.1	RAMSLTIEPRPVKQSR			38.2428570	27.6	4.2e-11
428.9924	4	0.045	RAMSLTIEPRPVKQS			45.6854540	25.9	7.3e-11
614.9983	3	3.0	ASRQGLTVSFIPYFKE			92.5646640	17.4	3.2e-9
614.2806	2	0.12	FVPGCFVCLE	Carbamidomethyl@5; Carbamidomethyl@8		108.28199	23.4	3.8e-9
658.0088	3	-2.9	ASRQGLTVSFIPYFKEE			93.2353870	25.4	1.2e-8
472.0164	4	0.043	RAMSLTIEPRPVKQSR		Oxidation@3	32.1087810	19.8	1.4e-8
690.8532	2	-1.1	SNKIYVVDLSNE			71.874420	27.2	3.9e-8
541.7657	2	-2.2	VDTYRPFQG			52.6320720	19.1	3.5e-7
678.3301	2	3.1	KFVPGCFVCLE	Carbamidomethyl@6; Carbamidomethyl@9		88.1169730	18.2	3.5e-7

b **PL45 CDCP1 Peptide Coverage**

1 MAGLNCGVSI ALLGVLLLGA ARLPRGAEAF EIALPRESNI TVLIKLGTPT LLAKPCYIVI SKRHITMLSI KSGERIVFTF
81 SCQSPENHFV IE**IQKNIDCM SGPCPFGE**VQ LQPSTSLLPT LNRTFIWDVK AHKSIGLELQ FSIPRLRQIG PGESCPDGVT
161 HSISGRIDAT VVRIGTFCSN GTVSRIKMQE GVKMALHLPW FHPRNVSGFS IANRSSIKRL CIIESVFEGE GSATLMSANY
241 PEGFPEDELM TWQFVVPAHL RASVSFLNFN LSNCERKEER VEYYIPGSTT NPEVFKLEDK QPGNMAGNFN LSLQGCDQDA
321 QSPGILRLQF QVLVQHPQNE **SNKIYVVDLS NERAMSLTIE PRPVKQSRKF VPGCFVCLE**S RTCSSNLTLT SGSKHKISFL
401 CDDLTRLWMN VEKTISCTDH RYCQRKSYSL QVPSDILHLP VELHDFSWKL LVPKDRLSLV LVPAQKLQQH THEKPCNTSF
481 SYLVASAIPS QDLYFGSFCP GGSIKQIQVK QNISVTLRTF APSFQQE**ASR QGLTVSFIPY FKEE**GVFTVT PDTKSKVYLR
561 TPNWDRGLPS LTSVSWNISV PRDQVACLTF FKERSGVVCQ TGRAFMIIQE QRTRAEEIFS LDEDVLPKPS FHHHSFWVNI
641 SNCSPTSGKQ LDLLFSVTLT PRTVDLTVIL IAAVGGGVLL LSALGLIICC VKKKKKKTNK GPAVGIYNDN INTEMPRQPK
721 KFQKGRKDND SHVYAVIEDT MVYGHLLQDS SGSFLQPE**VD TYRPFQG**TMG VCPPSPPTIC SRAPTAKLAT EEPPPRSPPE
801 SESEPYTFSH PNNGDVSSKD TDIPLLNTQE PMEPAE

m/z	z	ppm	DB Peptide	Constant Mods	Variable Mods	RT	Score	Expect
926.9037	2	-1.4	IQKNIDCMSGPCPFGE	Carbamidomethyl@7; Carbamidomethyl@12		73.341615	32.6	8.9e-13
499.9567	3	-0.92	RAMSLTIEPRPVK			45.8198360	26.9	1.6e-11
468.0182	4	1.1	RAMSLTIEPRPVKQSR			38.2428570	27.6	4.2e-11
428.9924	4	0.045	RAMSLTIEPRPVKQS			45.6854540	25.9	7.3e-11
614.9983	3	3.0	ASRQGLTVSFIPYFKE			92.5646640	17.4	3.2e-9
614.2806	2	0.12	FVPGCFVCLE	Carbamidomethyl@5; Carbamidomethyl@8		108.28199	23.4	3.8e-9
658.0088	3	-2.9	ASRQGLTVSFIPYFKEE			93.2353870	25.4	1.2e-8
472.0164	4	0.043	RAMSLTIEPRPVKQSR		Oxidation@3	32.1087810	19.8	1.4e-8
690.8532	2	-1.1	SNKIYVVDLSNE			71.874420	27.2	3.9e-8
541.7657	2	-2.2	VDTYRPFQG			52.6320720	19.1	3.5e-7
678.3301	2	3.1	KFVPGCFVCLE	Carbamidomethyl@6; Carbamidomethyl@9		88.1169730	18.2	3.5e-7

c **HPAC CDCP1 Peptide Coverage**

1 MAGLNCGVSI ALLGVLLLGA ARLPRGAEAF EIALPRESNI TVLIKLGTPT LLAKPCYIVI SKRHITMLSI KSGERIVFTF
81 SCQSPENHFV IE**IQKNIDCM SGPCPFGE**VQ LQPSTSLLPT LNRTFIWDVK AHKSIGLELQ FSIPRLRQIG PGESCPDGVT
161 HSISGRIDAT VVRIGTFCSN GTVSRIKMQE GVKMALHLPW FHPRNVSGFS IANRSSIKRL CIIESVFEGE GSATLMSANY
241 PEGFPEDELM TWQFVVPAHL RASVSFLNFN LSNCERKEER VE**YYIPGSTT NPE**VFKLEDK QPGNMAGNFN LSLQGCDQDA
321 QSPGILRLQF QVLVQHPQNE **SNKIYVVDLS NERAMSLTIE PRPVKQSRKF VPGCFVCLE**S RTCSSNLTLT SGSKHKISFL
401 CDDLTRLWMN VEKTISCTDH RYCQRKSYSL QVPSDILHLP VELHDFSWKL LVPKDRLSLV LVPAQKLQQH THEKPCNTSF
481 SYLVASAIPS QDLYFGSFCP GGSIKQIQVK QNISVTLRTF APSFQQEASR QGLTVSFIPY FKEEGVFTVT PDTKSKVYLR
561 TPNWDRGLPS LTSVSWNISV PRDQVACLTF FKE**RSGVVCQ TGRAFMIIQE** QRTRAEEIFS LDEDVLPKPS FHHHSFWVNI
641 SNCSPTSGKQ LDLLFSVTLT PRTVDLTVIL IAAVGGGVLL LSALGLIICC VKKKKKKTNK GPAVGIYNDN INTEMPRQPK
721 KFQKGRKDND SHVYAVIEDT MVYGHLLQDS SGSFLQPEVD TYRPFQGTMG VCPPSPPTIC SRAPTAKLAT EEPPPRSPPE
801 SESEPYTFSH PNNGDVSSKD TDIPLLNTQE PMEPAE

m/z	z	ppm	DB Peptide	Constant Mods	Variable Mods	RT	Score	Expect
926.9043	2	-0.67	IQKNIDCMSGPCPFGE	Carbamidomethyl@7; Carbamidomethyl@12		73.4511430	29.8	1.2e-10
571.6540	3	-0.087	RAMSLTIEPRPVKQS			45.89985	22.9	2.9e-9
690.8530	2	-1.3	SNKIYVVDLSNE			71.9418880	26.0	2.1e-8
802.1774	4	2.6	RAMSLTIEPRPVKQSRKFVPGC FVCLE	Carbamidomethyl@22 ;Carbamidomethyl@2 5		77.273185	17.9	5.3e-6
621.2883	2	0.70	YYIPGSTTNPE			69.5404680	15.7	1.0e-5
651.3331	3	0.38	RSGVVCQTGRAFMIIQE	Carbamidomethyl@6		72.552615	15.7	5.8e-5

Supplementary Figure 3: CDCP1 peptides identified by IP-MS of PDAC cell lines. Peptides identified by IP-MS of 3 different PDAC cell lines (a) PL5, (b) PL45, (c) HPAC. Peptides corresponding to cleaved fragments of CDCP1 were identified on PL5 and PL45 cells, but not HPAC.

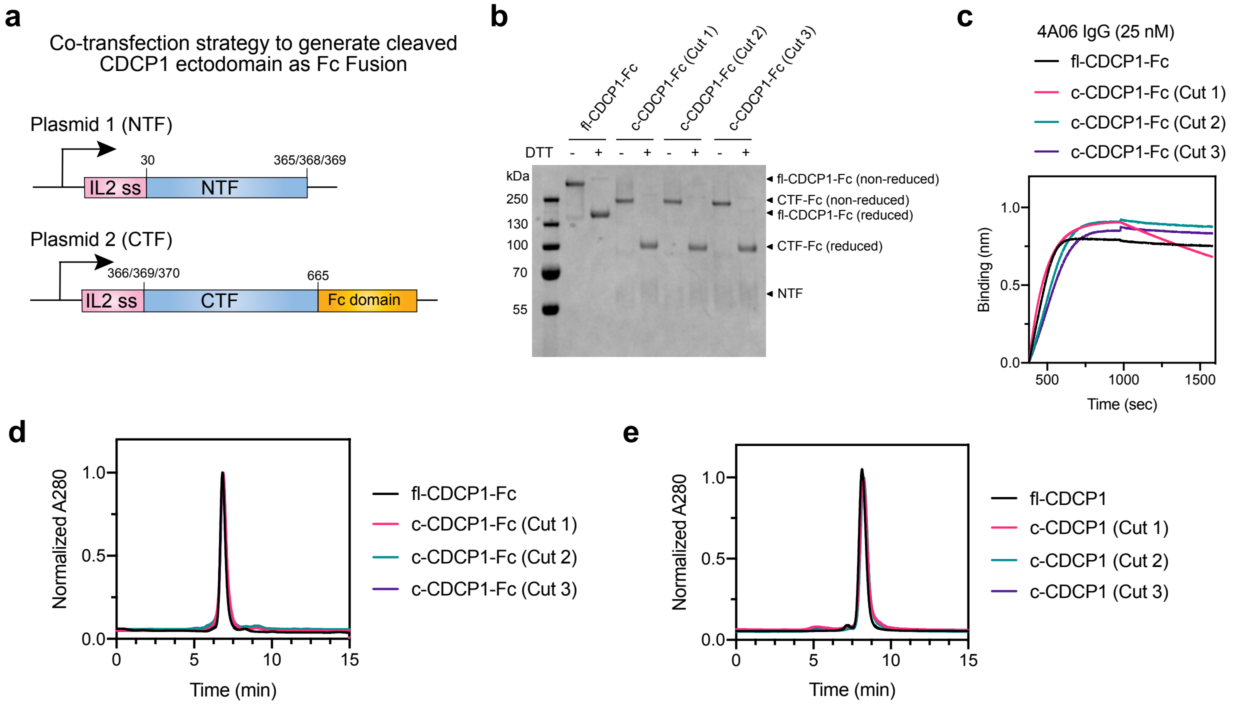

Supplementary Figure 4: Generation of fl-CDCP1 and c-CDCP1 ectodomain with endogenous cut sites. (**a**) Schematic of a two-plasmid co-transfection strategy to generate the cleaved CDCP1 ectodomain as an Fc fusion. The NTF and CTF-Fc were encoded on separate plasmids, with an IL2 signal sequence preceding each sequence. (**b**) SDS-PAGE gel of fl-CDCP1-Fc and c-CDCP1-Fc (Cut 1, Cut 2, Cut 3) (**c**) BLI shows robust binding of IgG 4A06 to fl-CDCP1-Fc and c-CDCP1-Fc. (**c**) BLI of IgG 4A06 to fl-CDCP1-Fc and c-CDCP1-Fc (Cut 1, Cut 2, Cut 3) show robust binding, indicating NTF is present on all 4 constructs. (**d**) SEC of fl-CDCP1-Fc and c-CDCP1-Fc (Cut 1, Cut 2, Cut 3) shows that all 4 antigens have similar elution profiles. (**e**) SEC of fl-CDCP1 and c-CDCP1 (Cut 1, Cut 2, Cut 3) without Fc domains shows that all 4 antigens have similar elution profiles.

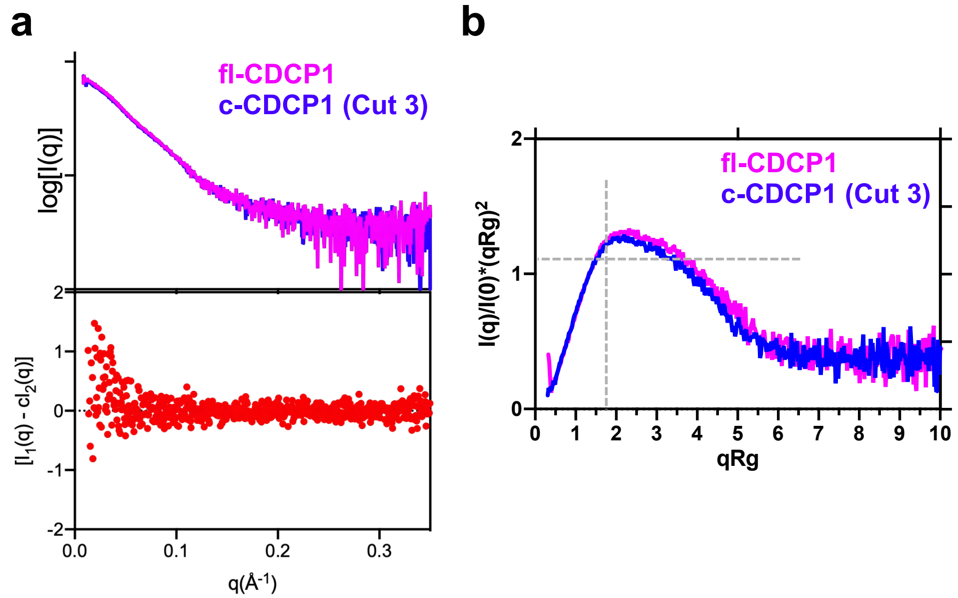

Supplementary Figure 5: SEC-SAXS of uncleaved and cleaved CDCP1 ectodomain show similar conformations. (**a**) SAXS profiles, (**b**) Normalized Kratky plot, and (**c**) P(r) function of fl-CDCP1 and c-CDCP1 (Cut 3) ectodomain indicates that there are no large-scale conformational changes between fl-CDCP1 and c-CDCP1.

Supplementary Table 1: SAXS and MALS experimental parameters

	R_g (Å)	Dmax (Å)	MW MALS/SAXS (kDa)
**fl-CDCP1**	45.4 +/- 0.6	145	97.0 (+/- 0.11%) / ~112
**c-CDCP1 (Cut3)**	44.1 +/- 0.5	143	99.6 (+/- 0.11%) / ~108

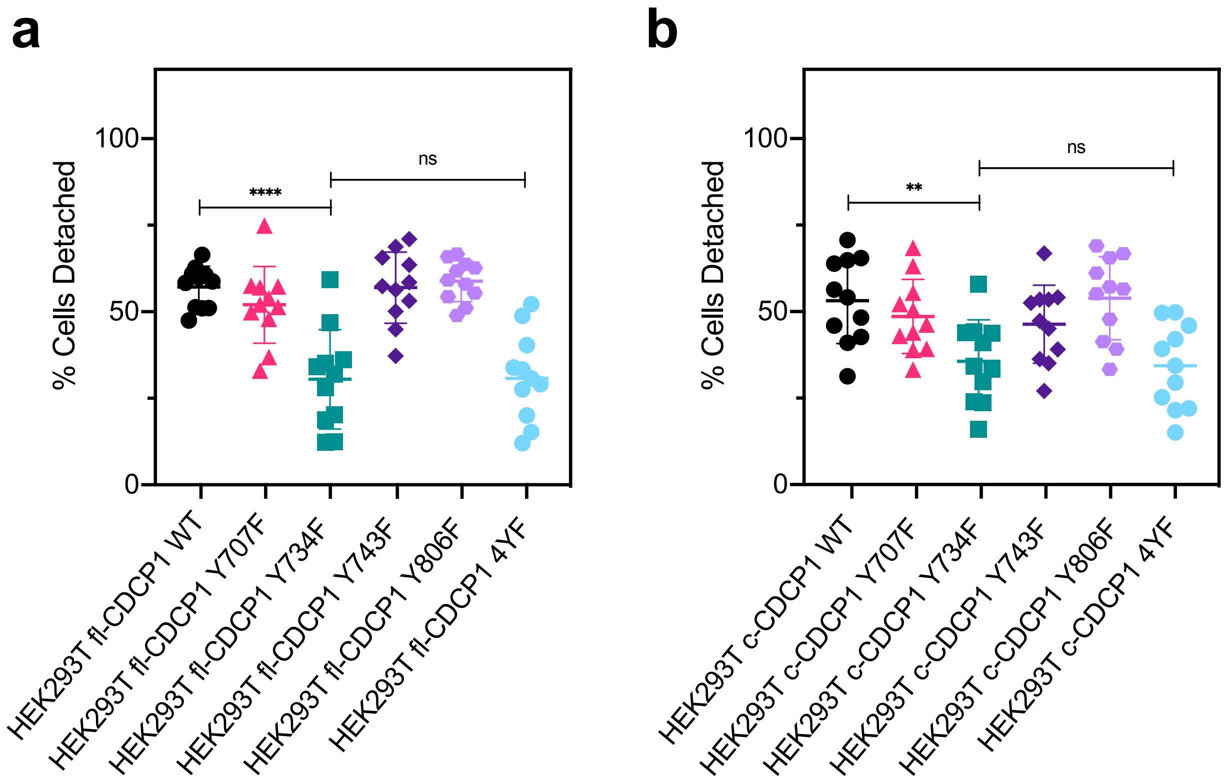

Supplementary Figure 6: Cell Adhesion Assay of HEK293T cells expressing CDCP1 tyrosine variants show that phosphorylation of Y734 is critical for detachment. Cell adhesion assay of for HEK293T cells expressing (**a**) fl-CDCP1 and (**b**) c-CDCP1 variants. The 4 intracellular tyrosine residues (Y707, Y734, Y743, Y806) were individually or altogether (4YF) mutated to phenylalanine to abrogate signaling. Data indicate that Y734 is important for CDCP1-associated intracellular signaling implicated in decreased cell adhesion. Data represent individual values and mean ± SEM. There was a significant difference in cell adhesion between the different cell lines expressing fl-CDCP1 variants ($F(6, 70) = 10.98$, $p < 0.0001$, one-way ANOVA) and c-CDCP1 variants ($F(5, 60) = 5.832$, $p < 0.0002$, one-way ANOVA). Tukey post-hoc tests revealed that the decreased cell adhesion resulting from overexpression of either fl-CDCP1 and c-CDCP1 was reversed when all 4 intracellular tyrosine residues (4YF) or specifically Y734F was mutated (****$p < 0.0001$ for fl-CDCP1 v. fl-CDCP1 Y734F, **$p = 0.011$ for c-CDCP1 v. c-CDCP1 Y734F). There was no significant difference between the Y734F and 4YF mutations, and there was no significant effect compared to wild-type when Y707, Y743, or Y806 was mutated ($p > 0.05$), suggesting that Y734 is the intracellular phosphorylation site responsible for modulating CDCP1-driven loss of cell adhesion.

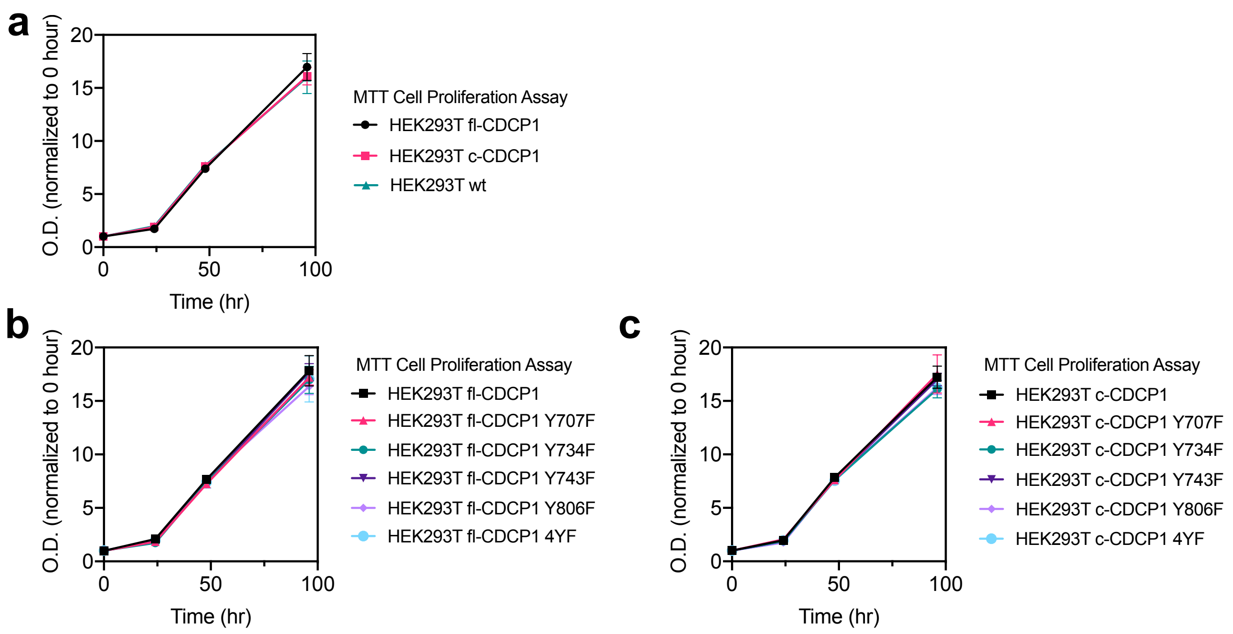

Supplementary Figure 7: MTT Cell proliferation assay of HEK293T cell lines expressing fl-CDCP1 and c-CDCP1 variants show over-expression, phosphorylation, or cleavage have no effect on cell proliferation. Cell proliferation assay measured by MTT for HEK293T cells expressing (**a**) HEK293T WT, fl-CDCP1, and c-CDCP1, (**b**) fl-CDCP1 variants, and (**c**) c-CDCP1 variants. fl-CDCP1 or c-CDCP1 overexpression does not have a significant effect on cell growth compared to HEK293T WT cells. All variants have similar rates of growth over a 4-day period. Data were collected in triplicate and average and standard deviation are shown.

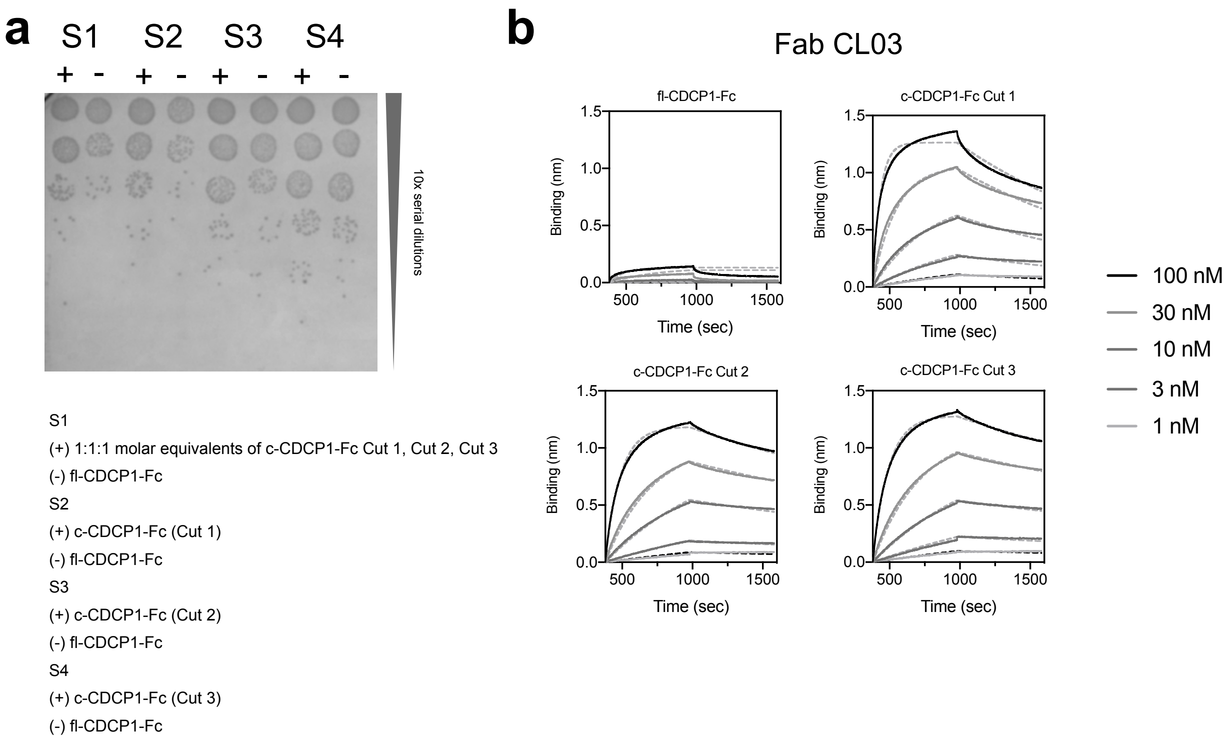

Supplementary Figure 8: Identification of cleaved CDCP1-specific Fab by phage selection. (**a**) Eluted phage from round 4 of phage selection indicate that there is enrichment for Fab-phage that recognize cleaved CDCP1 over uncleaved CDCP1. (**b**) BLI of Fab CL03 Fab binding to immobilized fl-CDCP1-Fc, c-CDCP1-Fc (Cut 1, Cut 2, Cut 3) shows that Fab CL03 selectively recognizes cleaved CDCP1 and not uncleaved CDCP1.

Supplementary Table 2: In vitro binding affinities of Fab CL03 and IgG CL03 to uncleaved and cleaved CDCP1

Ab	Antigen	K_d (nM)	k_a (sec^{-1} M^{-1})	k_d (sec^{-1})
Fab CL03	fl-CDCP1-Fc	n.d.	n.d.	n.d.
	c-CDCP1-Fc (Cut 1)	2.26 ± 0.03	(2.88 ± 0.03) x 10^5	(6.48 ± 0.05) x 10^{-4}
	c-CDCP1-Fc (Cut 2)	3.91 ± 0.02	(9.07 ± 0.03) x 10^5	(3.56± 0.02) x 10^{-4}
	c-CDCP1-Fc (Cut 3)	3.59 ± 0.02	(8.78 ± 0.03) x 10^5	(3.15 ± 0.02) x 10^{-5}
IgG CL03	fl-CDCP1-Fc	n.d.	n.d.	n.d.
	c-CDCP1-Fc (Cut 1)	0.84 ± 0.01	(6.40 ± 0.03) x 10^5	(5.35 ± 0.03) x 10^{-4}
	c-CDCP1-Fc (Cut 2)	0.37 ± 0.01	(4.73 ± 0.01) x 10^5	(1.74 ± 0.01) x 10^{-4}
	c-CDCP1-Fc (Cut 3)	0.15 ± 0.01	(4.92 ± 0.01) x 10^5	(7.43 ± 0.09) x 10^{-5}

n.d. = data not fit

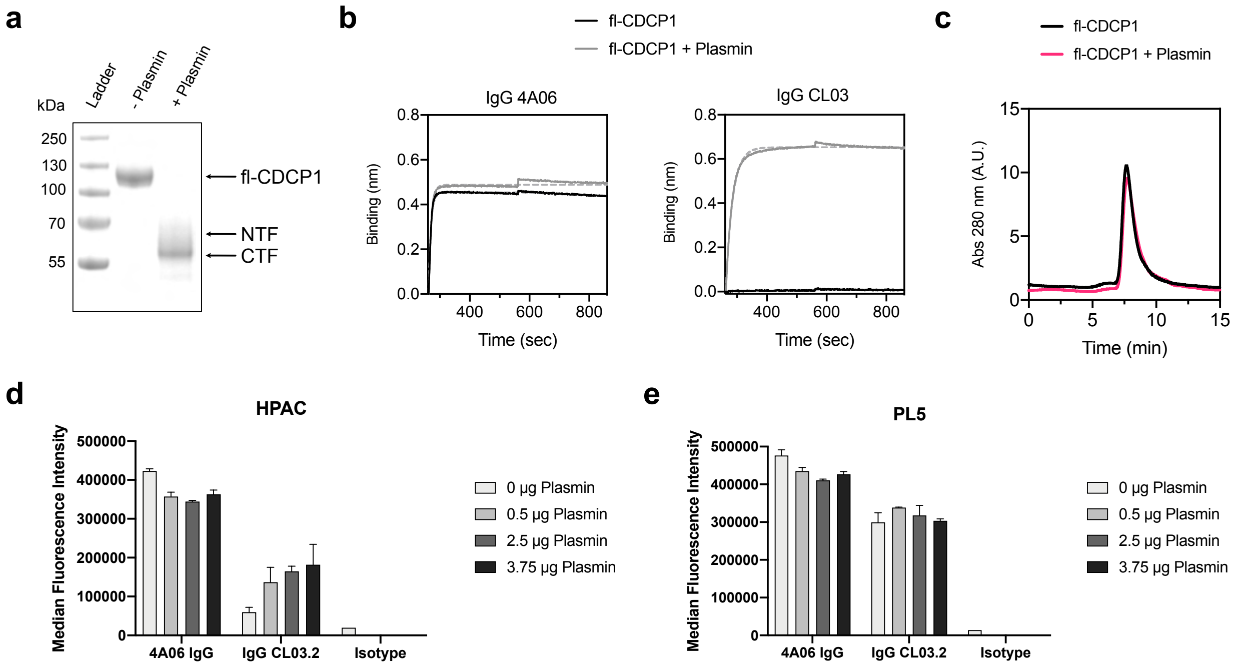

Supplementary Figure 9: IgG CL03 recognizes plasmin-cleaved CDCP1. (**a**) SDS-PAGE gel of fl-CDCP1 ectodomain treated with 0.5 µg plasmin indicates that plasmin can cleave CDCP1 at the expected molecular weights to generate c-CDCP1. (**b**) BLI of fl-CDCP1 treated with plasmin shows that IgG CL03 can specifically recognize plasmin-cleaved CDCP1. Binding of IgG 4A06 indicates that the NTF remains associated to the CTF. (**c**) SEC of plasmin-treated CDCP1 shows similar elution profiles to untreated fl-CDCP1. (**d**) Flow cytometry of HPAC cells treated with plasmin shows a dose-dependent increase in IgG CL03 binding, indicating that plasmin treatment can generate cleaved CDCP1 on the surface of HPAC cells. (**e**) Flow cytometry of PL5 cells treated with plasmin shows no additional increase in IgG CL03 binding.

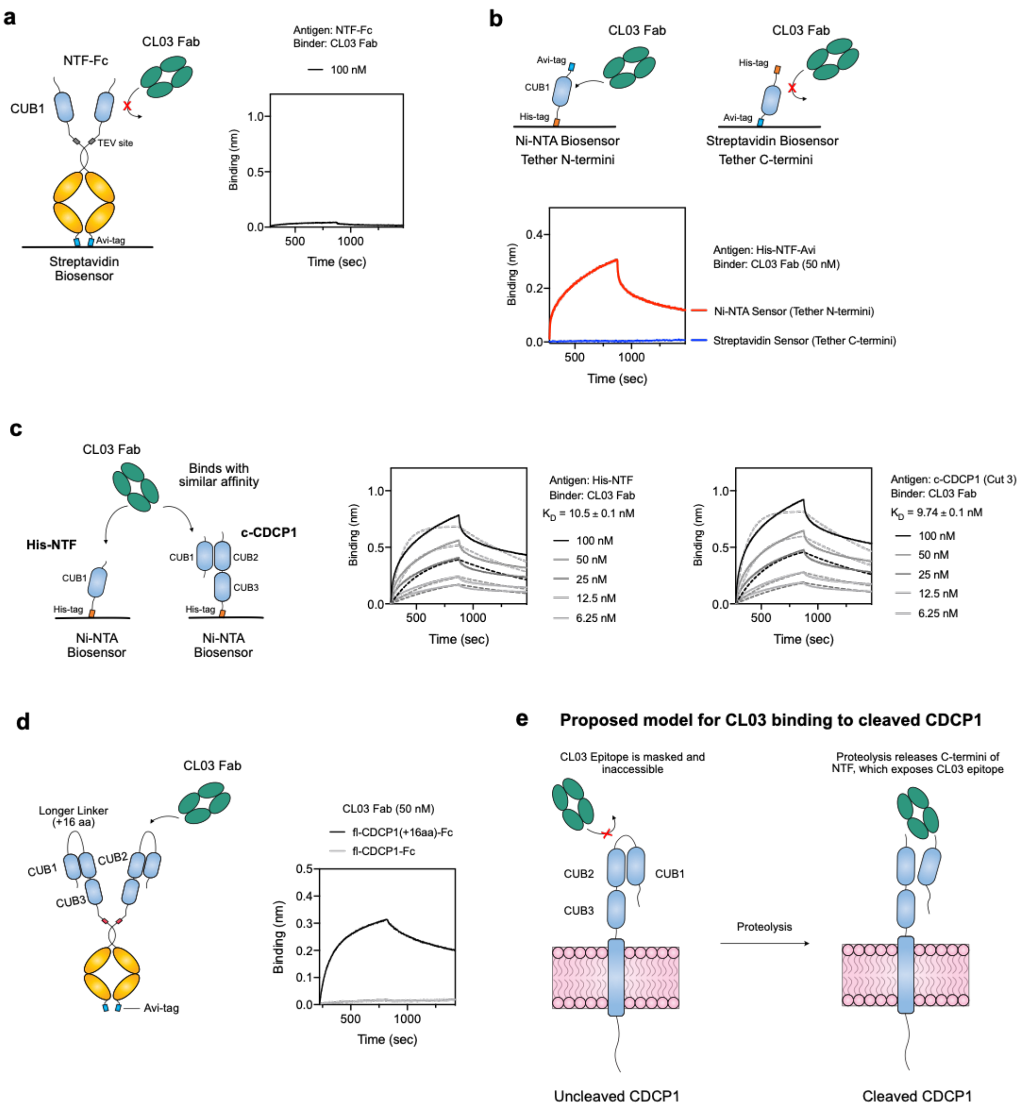

Supplementary Figure 10: CL03 binds to an epitope on the NTF that is exposed on cleaved CDCP1, but not uncleaved CDCP1. (**a**) BLI of CL03 Fab does not show binding to NTF-Fc. NTF-Fc was immobilized on a Streptavidin(SA) biosensor via a C-terminal biotinylated Avi-tag on the Fc domain. (**b**) BLI of CL03 Fab shows binding to N-terminally immobilized NTF, but not C-terminally immobilized NTF, indicating that an untethered C-termini of the NTF is necessary for CL03 binding. (**c**) Multipoint BLI show that CL03 Fab binds N-terminally tethered NTF at similar affinity to c-CDCP1, indicating the CL03 epitope is located within the NTF of CDCP1. (**d**) BLI shows CL03 Fab binding to uncleaved CDCP1 where the 2-residue R368/K369 cleavage site was replaced with a 16-amino acid linker. (**e**) Proposed model of CL03 binding to cleaved CDCP1. CL03 Fab recognizes an epitope on the NTF of CDCP1 that is masked and inaccessible when CDCP1 is uncleaved. Proteolysis releases the C-termini of the NTF and unmasks this epitope, allowing CL03 to bind and selectively recognize cleaved CDCP1 over uncleaved CDCP1.

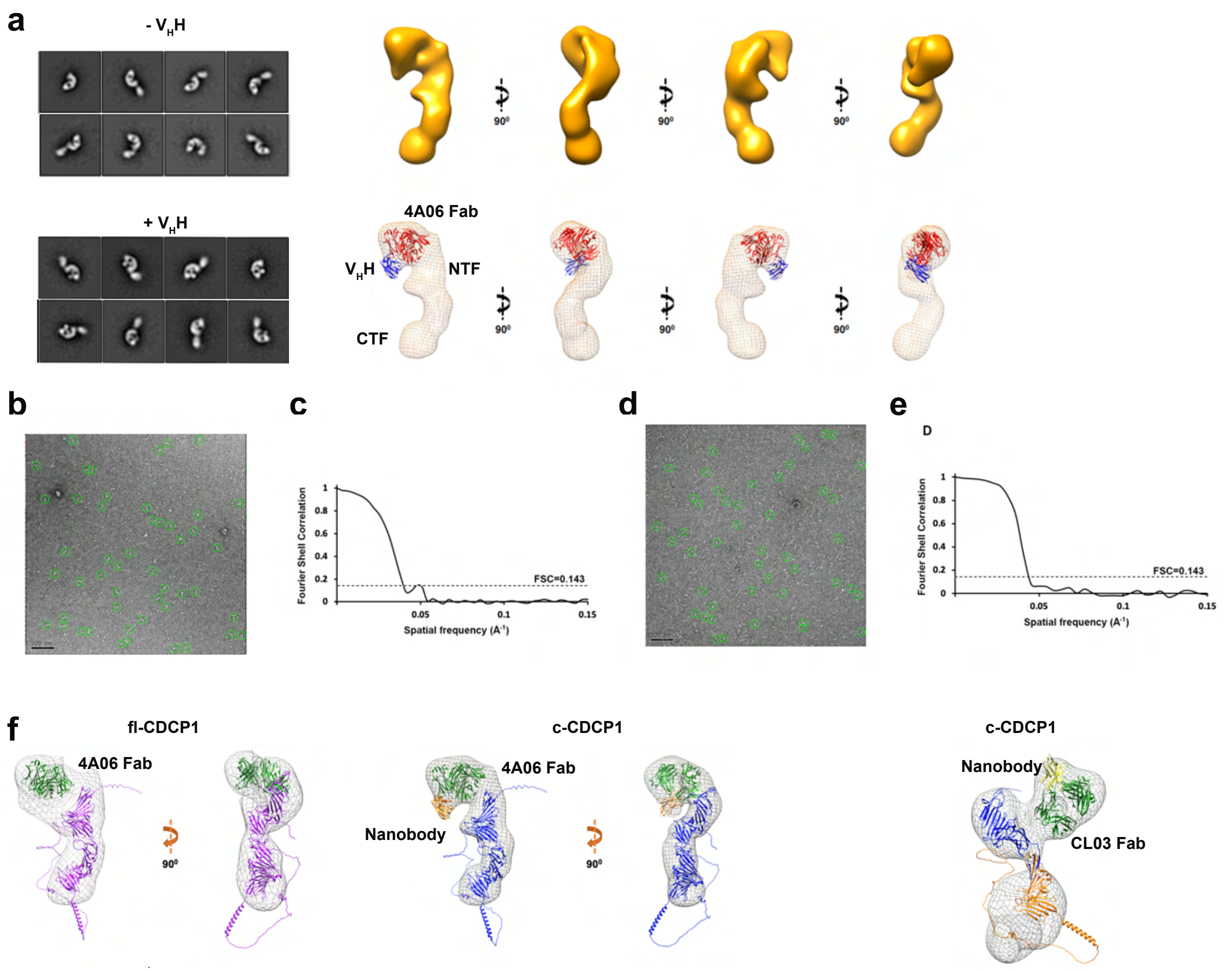

Supplementary Figure 11: Negative stain EM 3D reconstruction of cleaved CDCP1 bound to 4A06 Fab. (**a**) (*left*) 2D class averages of c-CDCP1(Cut3) + 4A06 Fab in the absence and presence of V_HH single domain antibody. (*right*) Different views of 3D negative stain EM map of c-CDCP1(Cut3) + 4A06 Fab + V_HH. Crystal structure of Fab (red) with V_HH (blue) were fitted into the 3D negative stain EM map. (**b**) A demonstrative micrograph of negatively stained c-CDCP1(Cut3) + CL03 Fab + V_HH particles. (**c**) A Fourier shell correlation plot used to determine the model resolution of 25 Å of c-CDCP1(Cut3) + CL03 Fab + V_HH, as given by 0.143 criterion. (**d**) A demonstrative micrograph of negatively stained c-CDCP1(Cut3) + 4A06 Fab + V_HH particles. (**e**) A Fourier shell correlation plot used to determine the model resolution of 23 Å of c-CDCP1(Cut3) + 4A06 Fab + V_HH, as given by 0.143 criterion. (**f**) The AlphaFold predicted model docked into the negative-stain electron microscopy maps of fl-CDCP1 (*left*) and c-CDCP1 (*middle*) bound to Fab 4A06-nanobody. The docking of the model to the EM map of c-CDCP1 bound to Fab CL03-nanobody (*right*) is less optimal, which could be due to the confirmation changes caused by Fab CL03 binding.

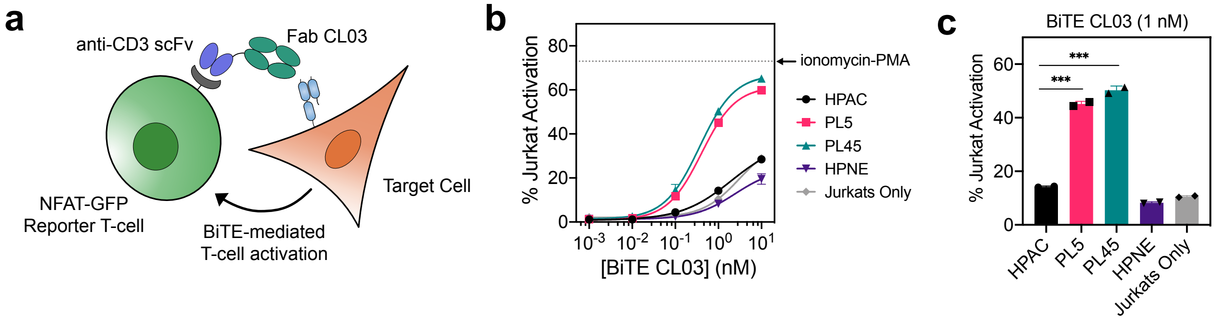

Supplementary Figure 12: CL03 as a Bi-specific T-cell engager (BiTE) can activate Jurkat cells in the presence of cleaved CDCP1-expressing PDAC cells. (**a**) Schematic of bi-specific T-cell engager (BiTE)-mediated T-cell activation assay. (**b**) Dose-dependent activation of NFAT-GFP reporter Jurkat cells above background were only observed in the presence of cleaved CDCP1-expressing PL5 and PL45 cells. ($n = 2$, error bars represent s.d.) (**c**) BiTE CL03 (1 nM) activates NFAT-GFP reporter T-cells in the presence of PL5 and PL45 cells but not HPAC or HPNE cells. (***$p<0.001$, unpaired T-test).

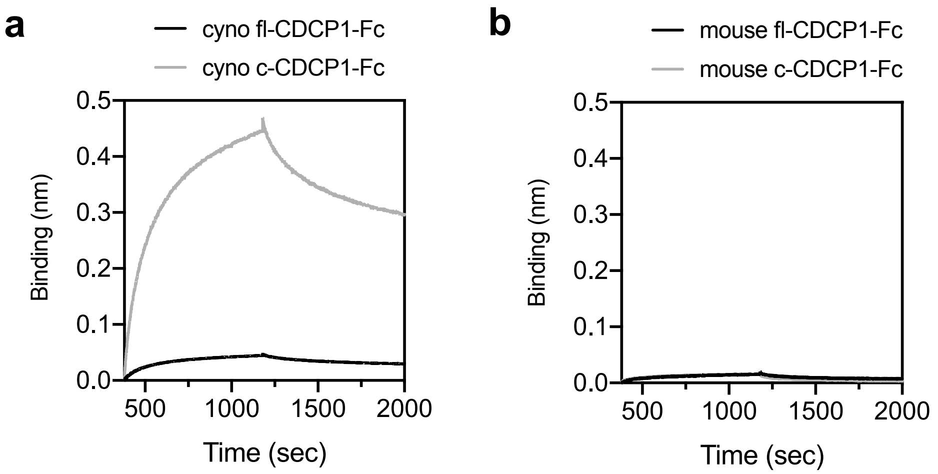

Supplementary Figure 13: Species cross-reactivity of IgG CL03. BLI of IgG CL03 to (**a**) cleaved cynomolgus CDCP1 homolog and (**b**) cleaved mouse CDCP1 homolog shows that IgG CL03 is cross-reactive and selective for cynomolgus cleaved CDCP1 but does not recognize mouse uncleaved or cleaved CDCP1.

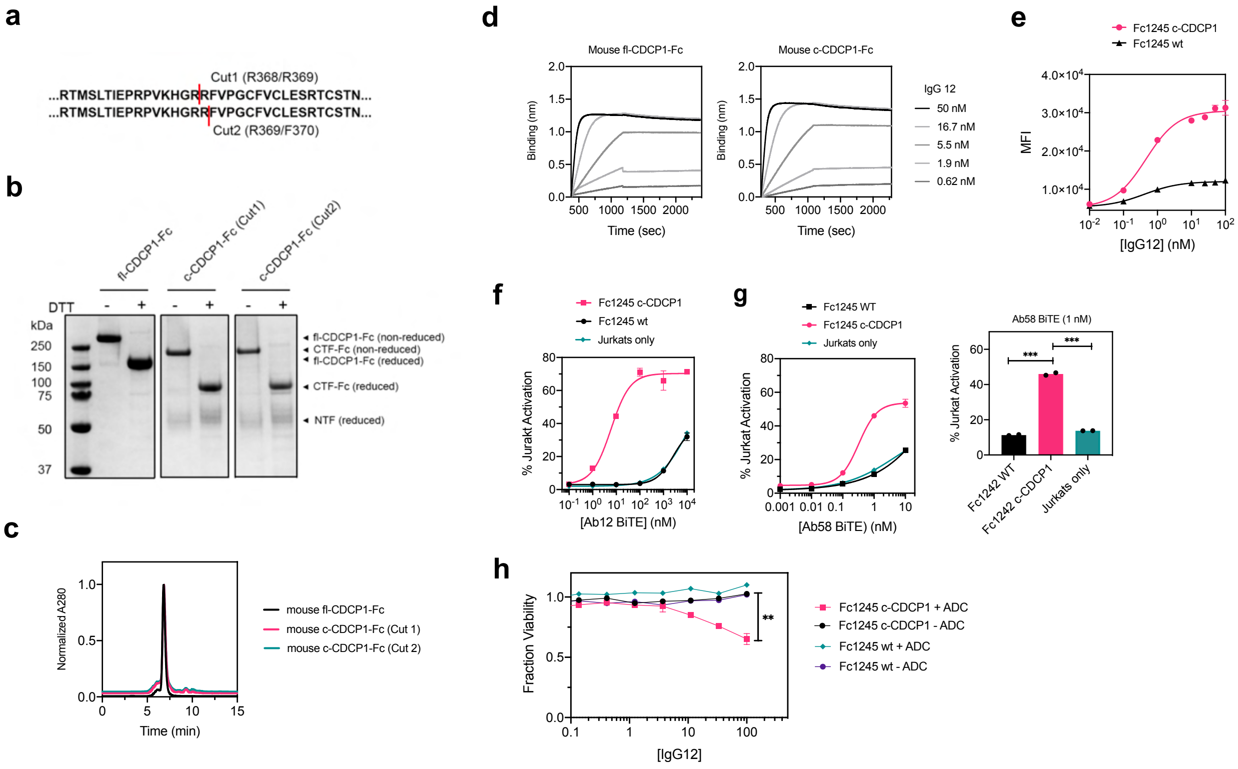

Supplementary Figure 14: Targeting mouse CDCP1 with IgG12 and IgG58. (**a**) Schematic of the two cut sites of cleaved mouse CDCP1. (**b**) SDS-PAGE gel of mouse CDCP1 antigens: fl-CDCP1-Fc, c-CDCP1-Fc (Cut 1), c-CDCP1-Fc (Cut 2) show proteins at the expected molecular weights. (**c**) SEC of mouse CDCP1 antigens: fl-CDCP1-Fc, c-CDCP1-Fc (Cut 1), c-CDCP1-Fc (Cut 2) show similar elution profiles, indicating the two fragments of cleaved CDCP1 is intact as a complex. (**d**) BLI of mouse CDCP1 antigens: fl-CDCP1-Fc and c-CDCP1-Fc show that IgG12, which recognizes the NTF of mouse CDCP1, can bind both cleaved and uncleaved mouse CDCP1, indicating that the NTF remains associated to the CTF. c-CDCP1-Fc was a 1:1 molar ratio of Cut 1 and Cut 2 antigens. (**e**) Flow cytometry shows that IgG58 binds robustly to Fc1245 c-CDCP1, and weakly to Fc1245 WT cells that express low levels of uncleaved CDCP1 (n = 3, error bars represent s.d.). (**f**) IgG12 reformatted to a BiTE shows dose-dependent activation of NFAT-GFP reporter Jurkat cells above background only in the presence of Fc1245 c-CDCP1 cells, but not in the presence of Fc1245 WT cells. (n = 2, error bars represent s.d.) (**g**) IgG58 reformatted to a BiTE shows dose-dependent activation of NFAT-GFP reporter Jurkat cells above background only in the presence of Fc1245 c-CDCP1 cells, but not in the presence of Fc1245 WT cells. (n = 2, error bars represent s.d.) (***p = 0.005, unpaired T-test) (**h**) Dose-dependent ADC-mediated cell killing with IgG12 and a secondary antibody conjugated to MMAF was only observed in Fc1245 c-CDCP1 cells and not Fc1245 WT cells. (n = 2, error bars represent s.d.) (**p = 0.0037, unpaired T-test)

Supplementary Table 3: Binding affinity of mouse CDCP1 antibodies to cleaved and uncleaved forms of mCDCP1

Ab	Antigen	K_d (nM)	k_a (sec^{-1} M^{-1})	k_d (sec^{-1})
IgG 12	fl-CDCP1-Fc	0.18 ± 0.01	(2.91 ± 0.01) x 10^5	(5.20 ± 0.10) x 10^{-5}
	c-CDCP1-Fc	0.16 ± 0.01	(2.82 ± 0.01) x 10^5	(4.54 ± 0.01) x 10^{-5}
IgG 58	fl-CDCP1-Fc	n.d.	n.d.	n.d.
	c-CDCP1-Fc	0.80 ± 0.01	(3.89 ± 0.01) x 10^5	(3.11 ± 0.06) x 10^{-5}

n.d. = data not fit

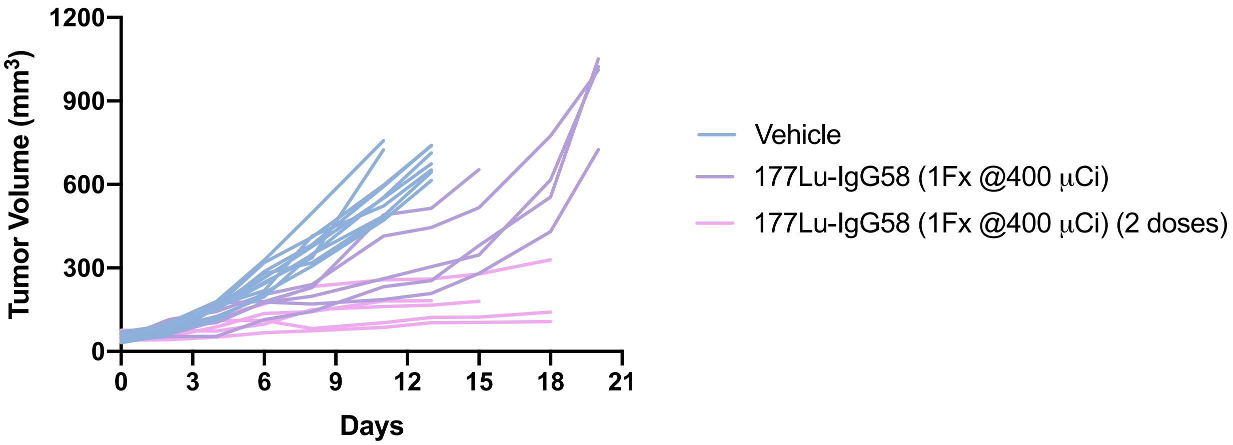

Supplementary Figure 15: Tumor volume of individual mice in ^{177}Lu-IgG58 theranostic study. Tumor volume of mice treated with vehicle or one or two 400 μCi doses of ^{177}Lu-IgG58 (n = 8 for vehicle, n = 5 per treatment arm) were monitored daily.

full unedited gel for Fig. 4D

replicate 1

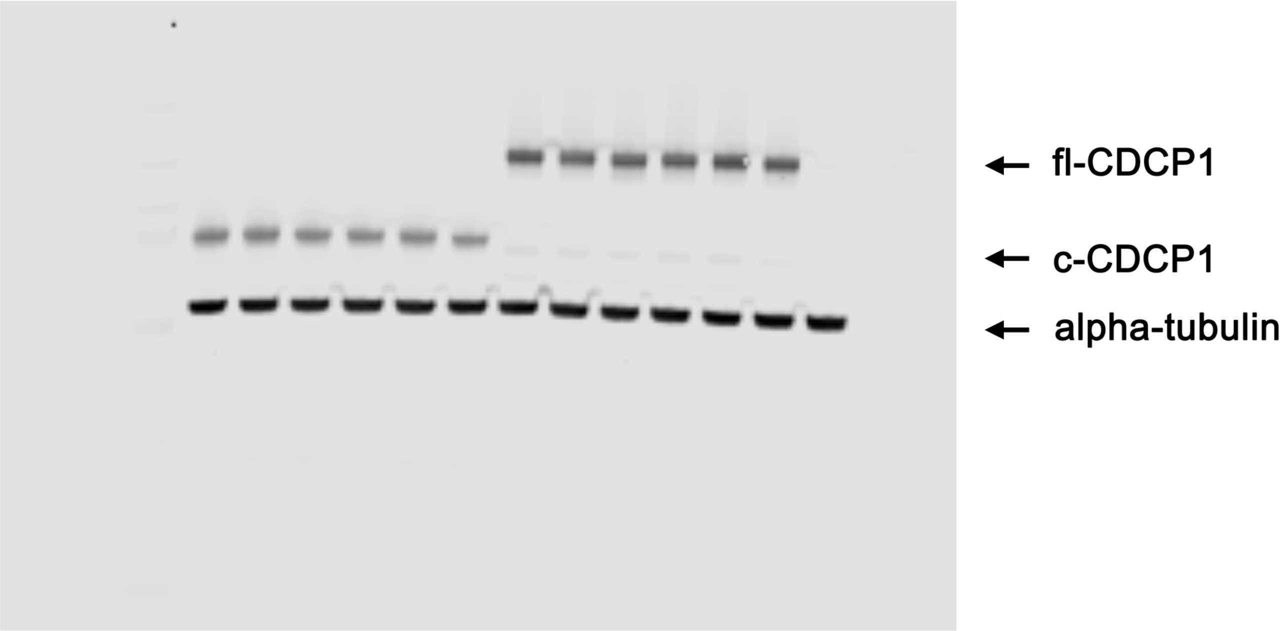

replicate 2

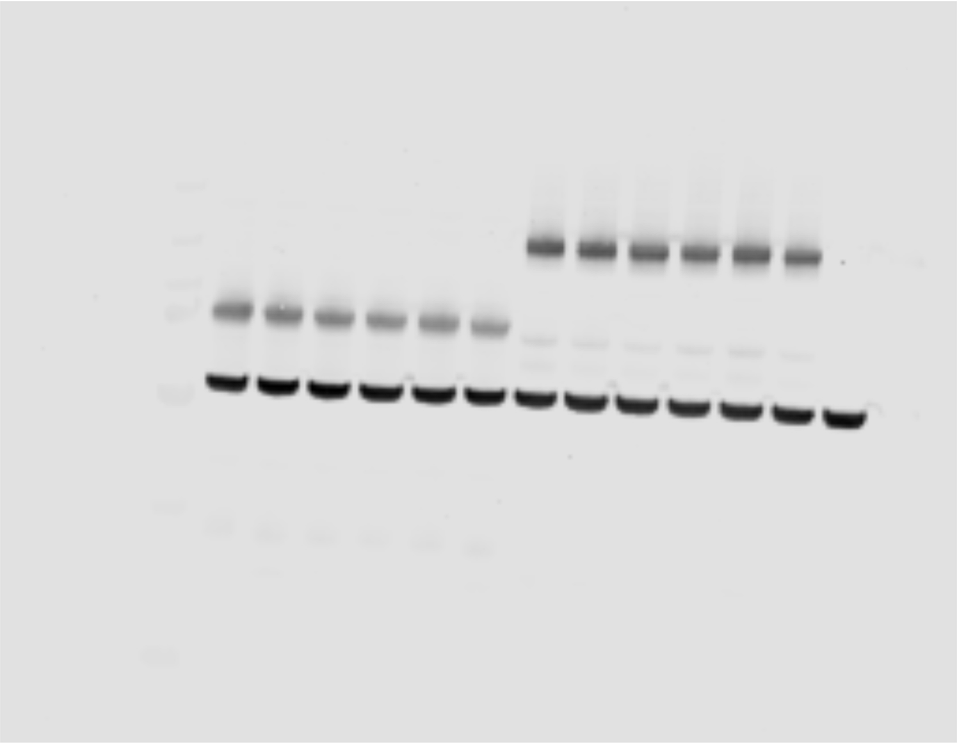

full unedited gel for Fig. 4D

replicate 1

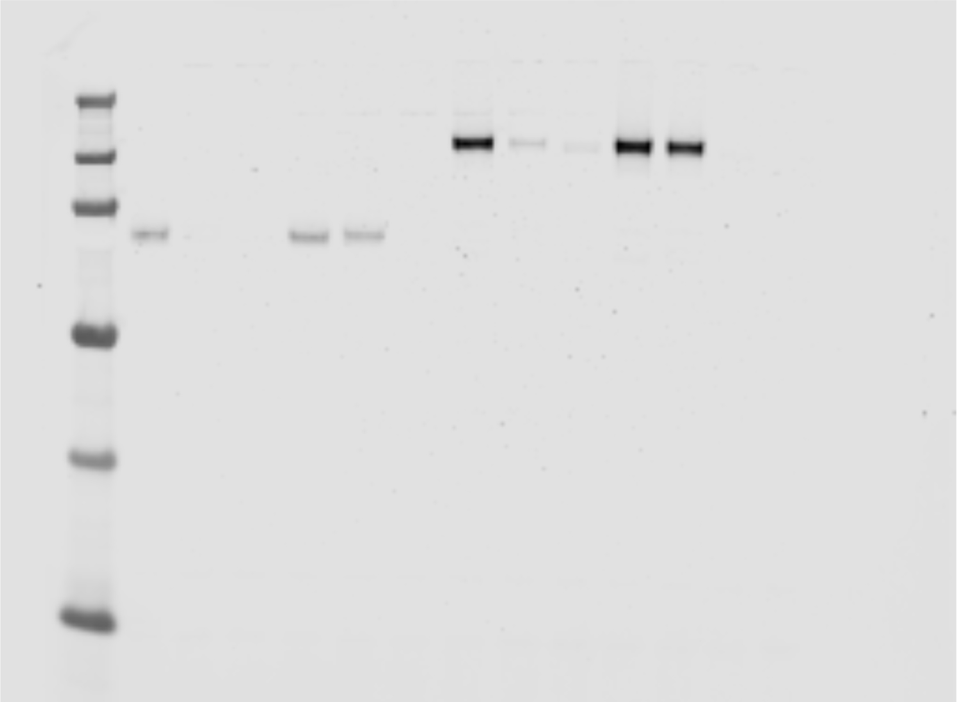

← fl-CDCP1 (pY707)

← c-CDCP1 (pY 707)

replicate 2

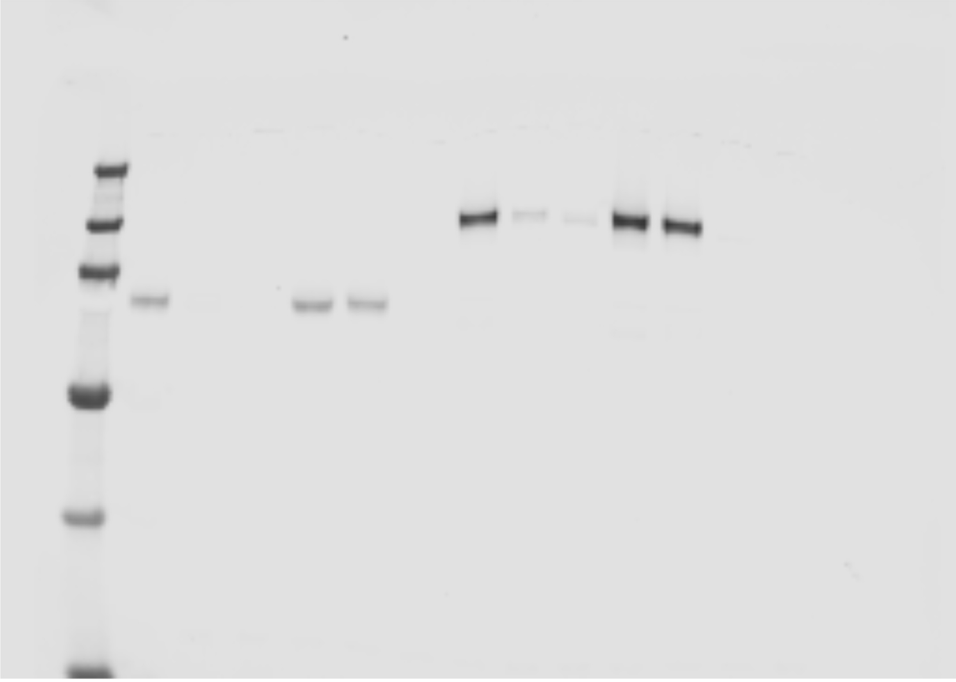

full unedited gel for Fig. 4D

replicate 1

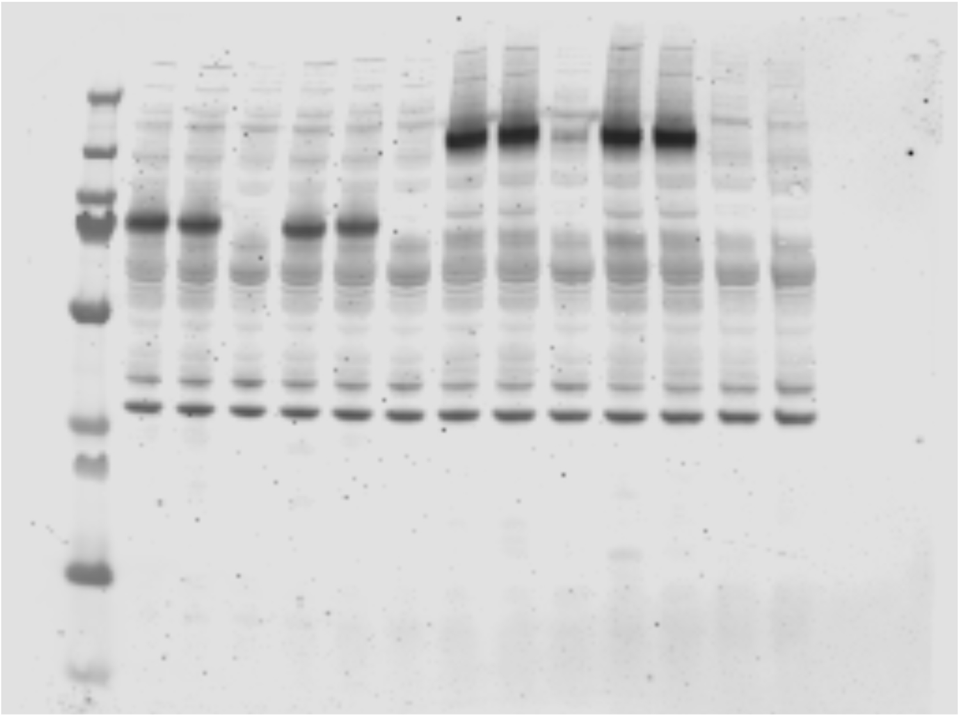

pan-pY (4G10)

replicate 2

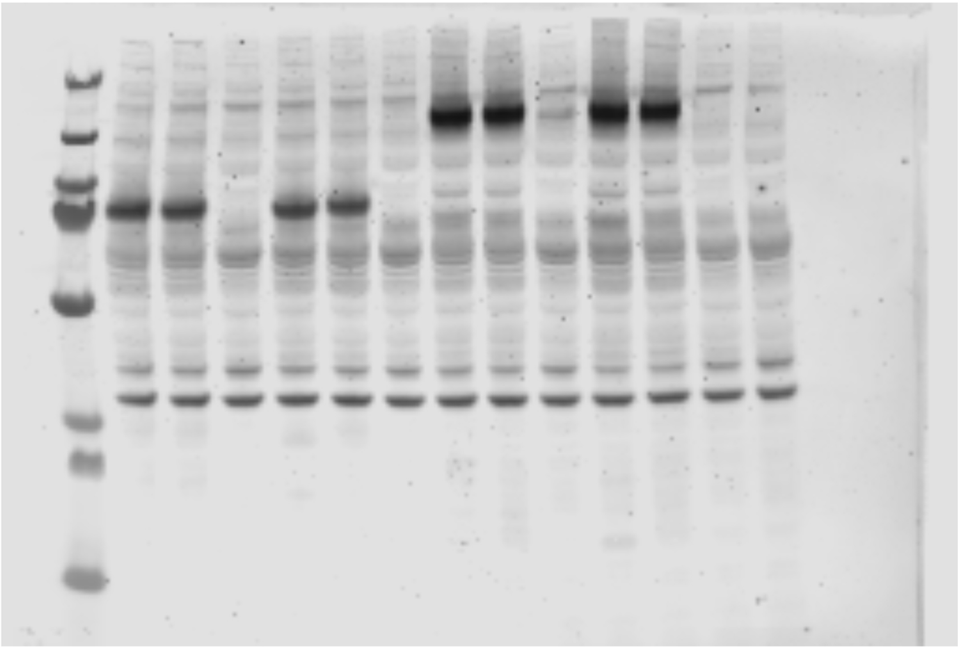

full unedited gel for Fig. 4D

replicate 1

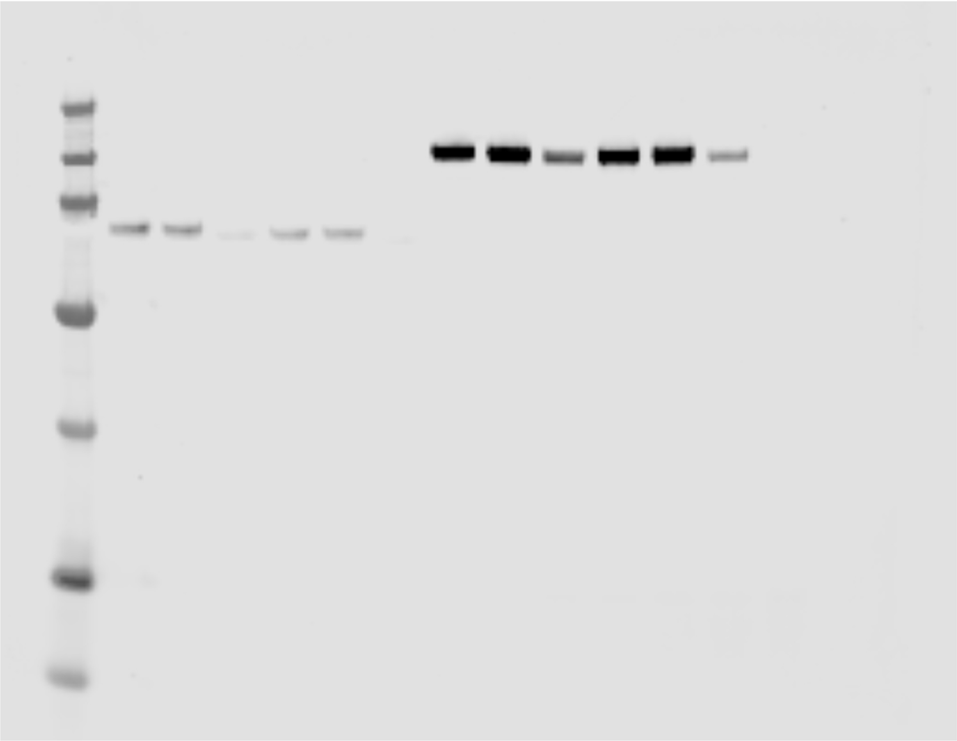

replicate 2

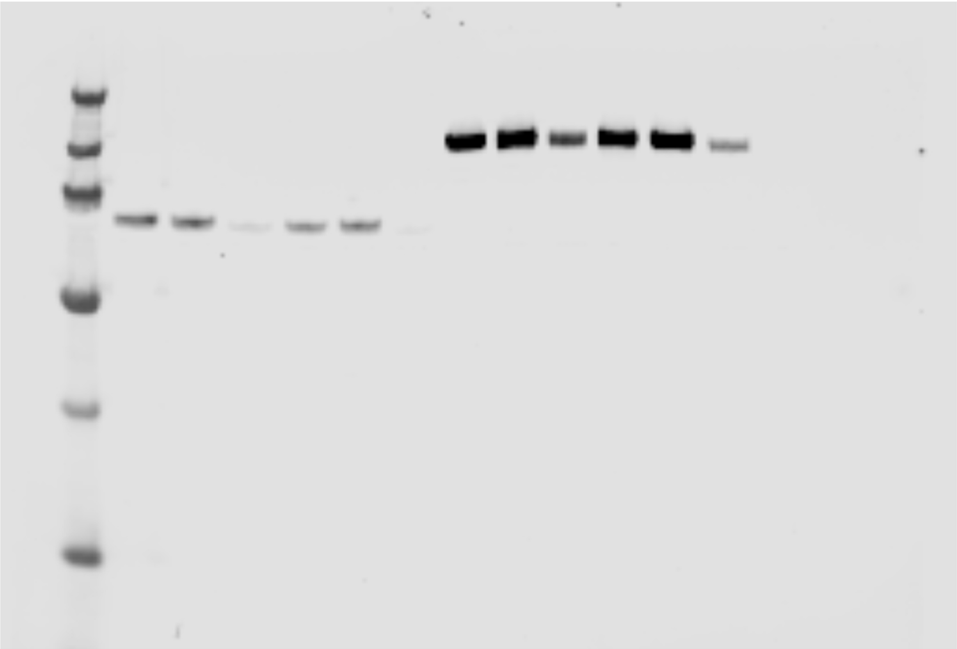

full unedited gel for Fig. 4D

replicate 1

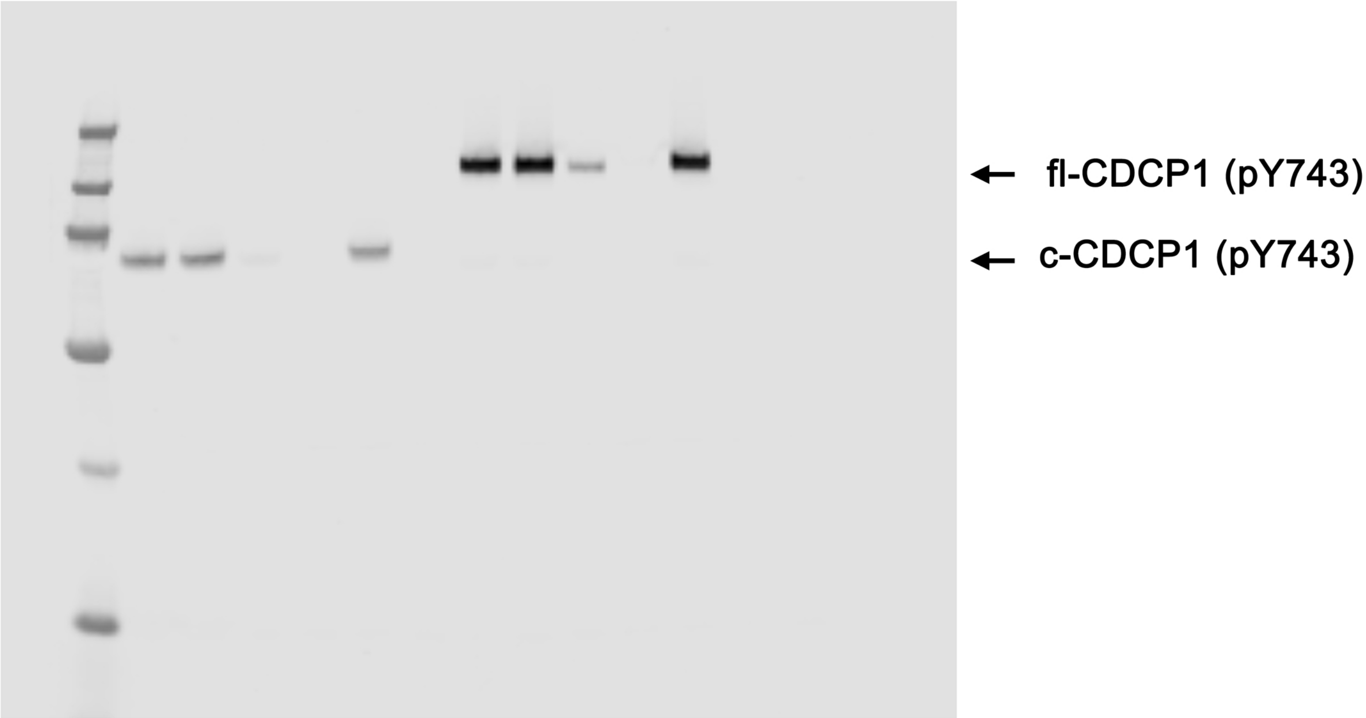

replicate 2

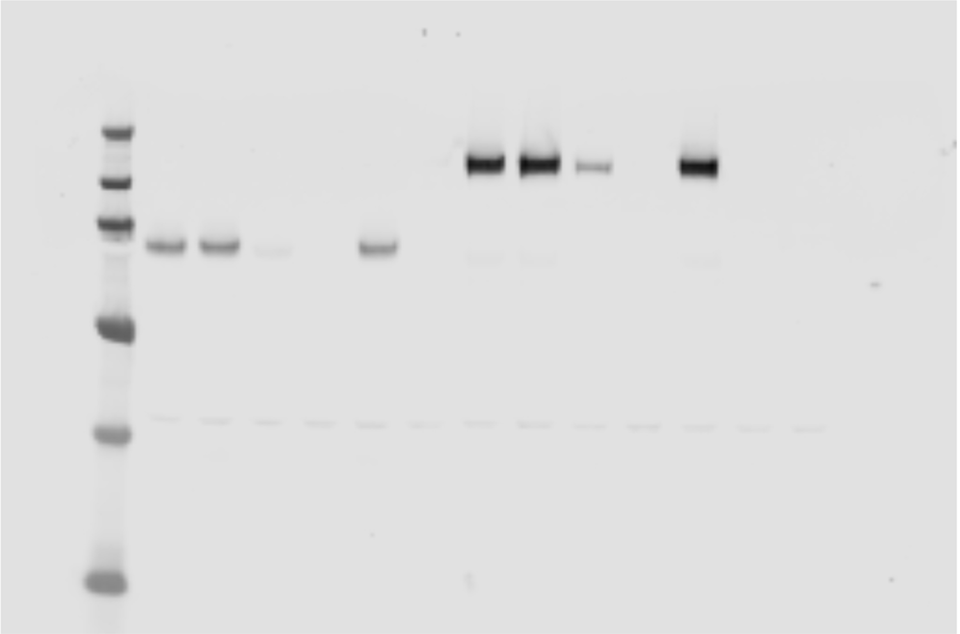

full unedited gel for Fig. 4D

replicate 1

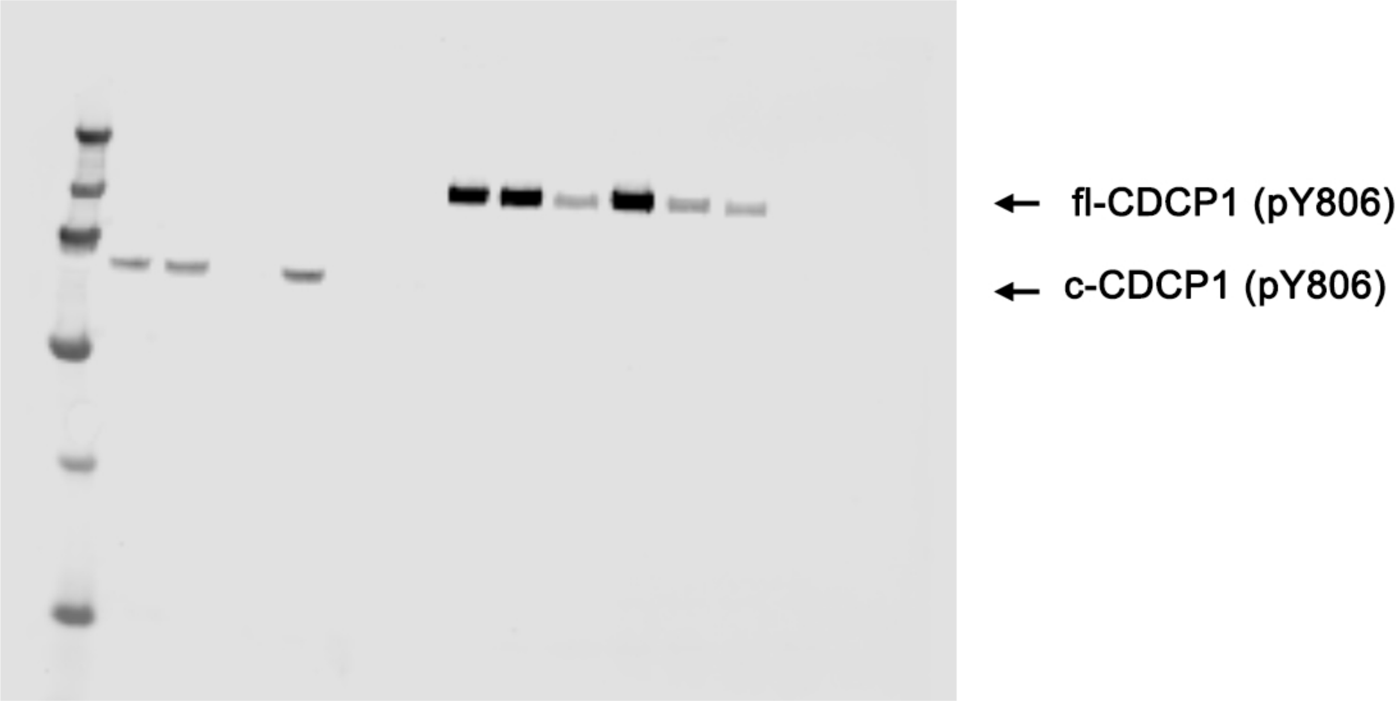

replicate 2

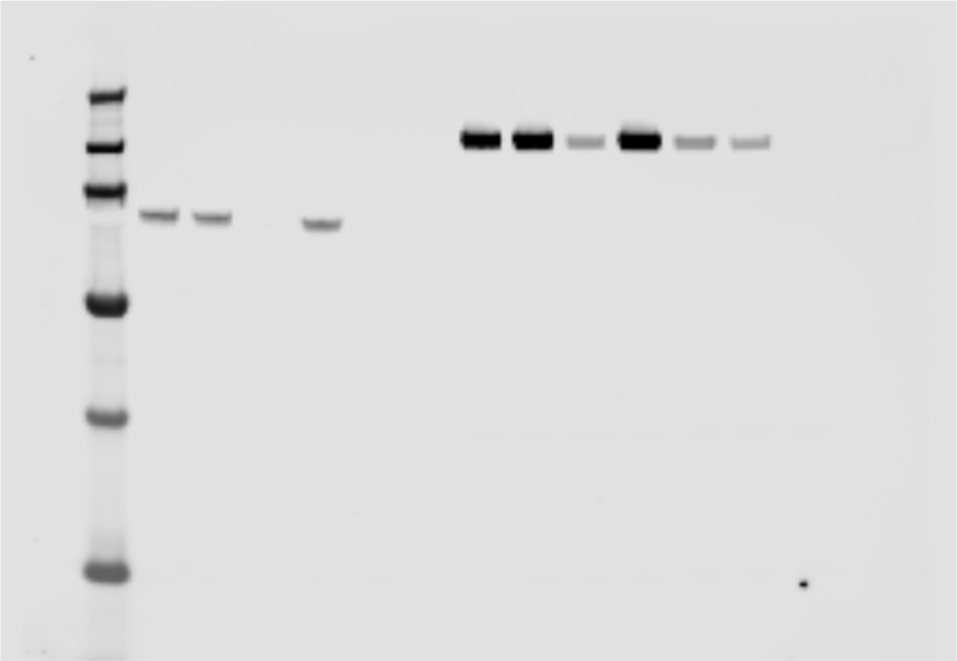

full unedited gel for Fig. 4D

replicate 1

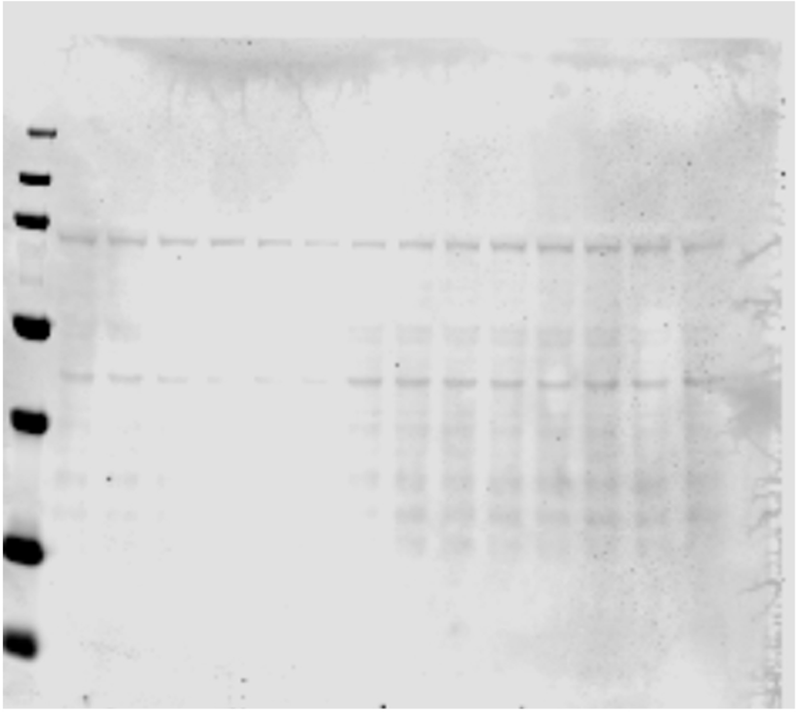

replicate 2

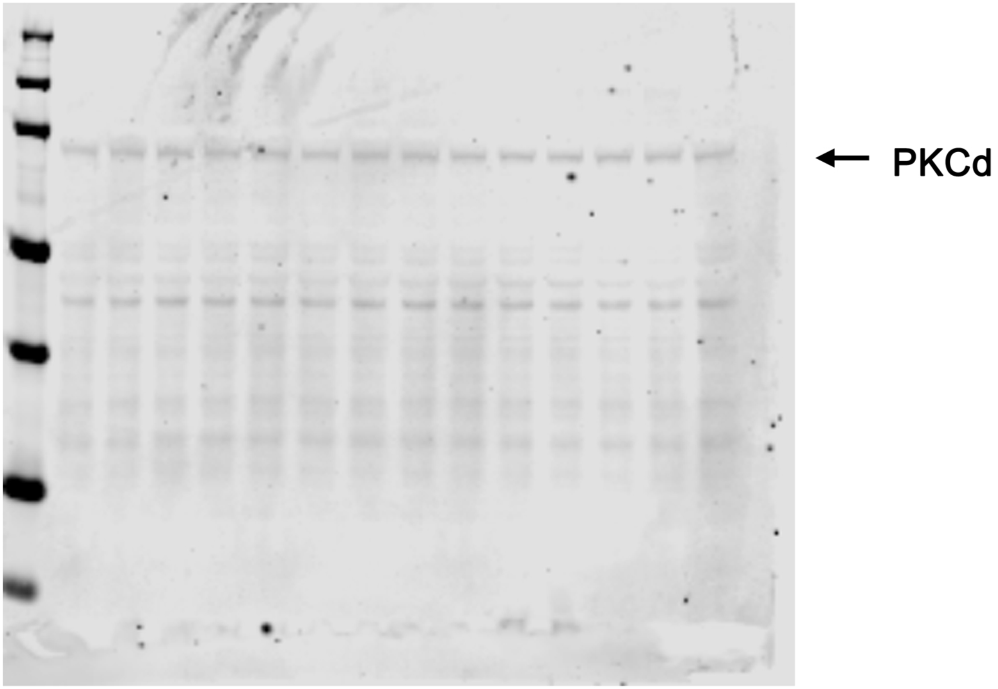

full unedited gel for Fig. 4D

replicate 1

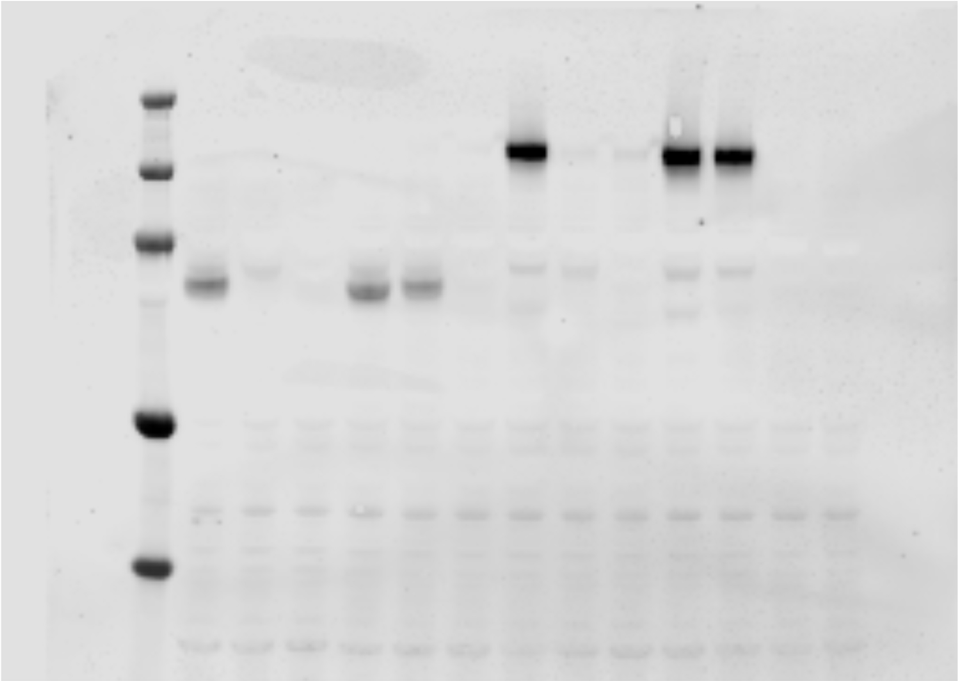

← PKCd (pY311)

replicate 2

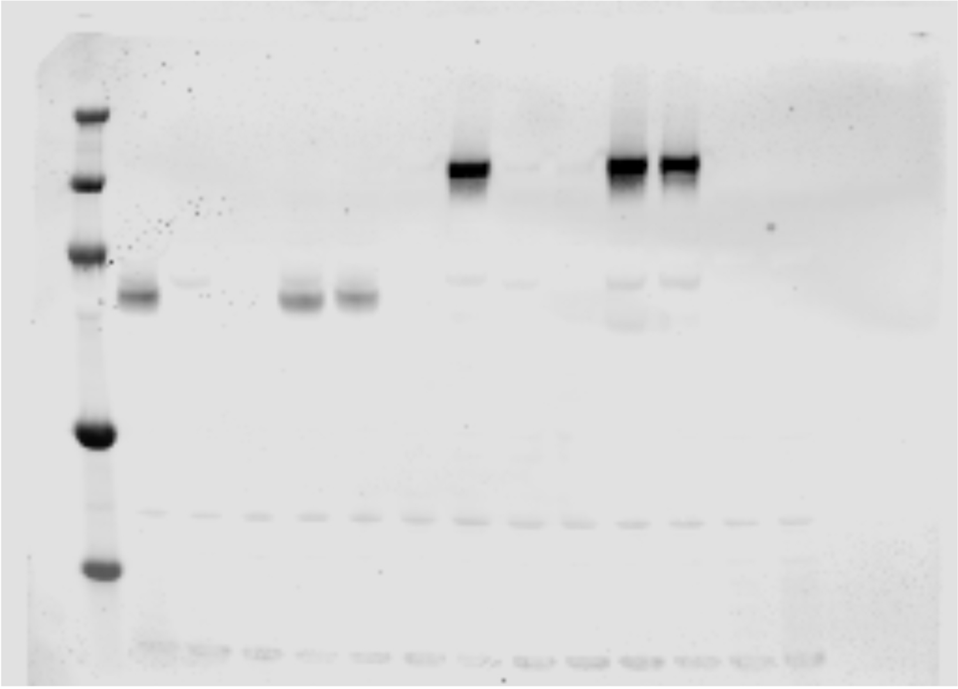

full unedited gel for Fig. 4D

replicate 1

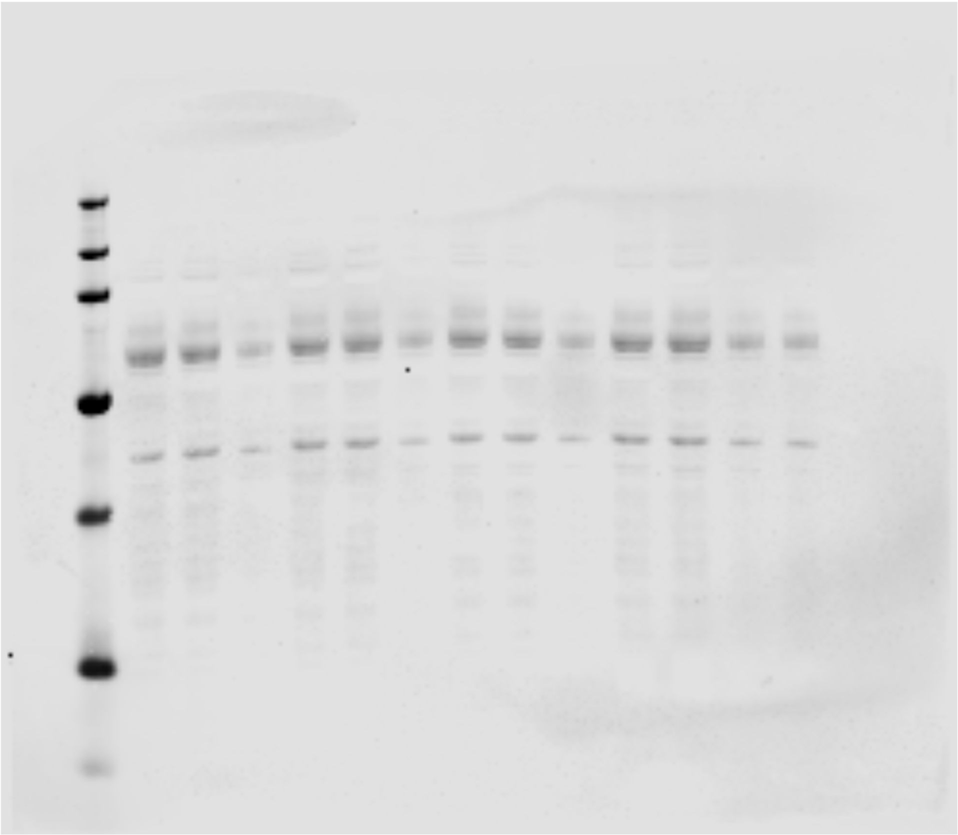

← SRC （pY416）

replicate 2

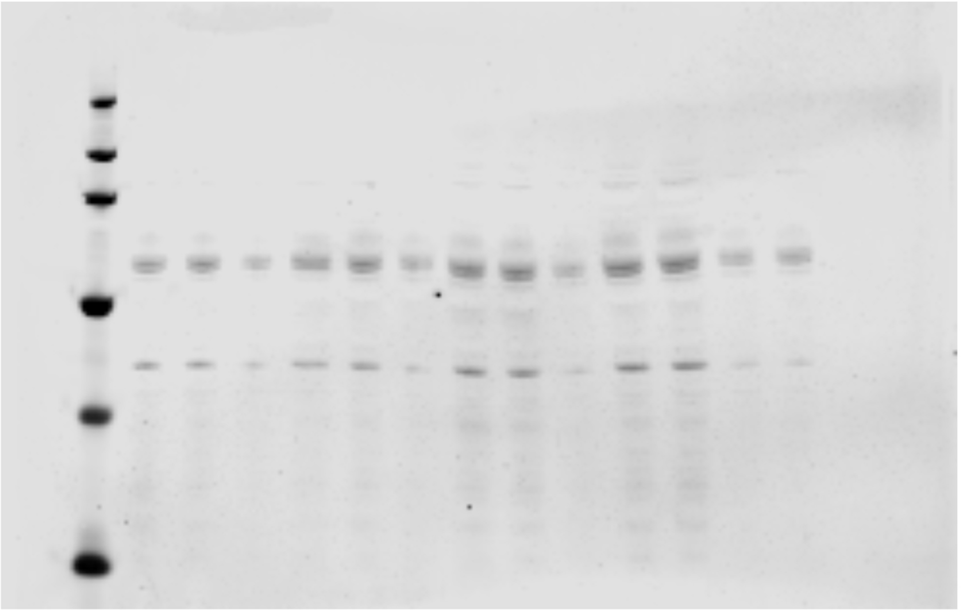

full unedited gel for Fig. 4D

replicate 1

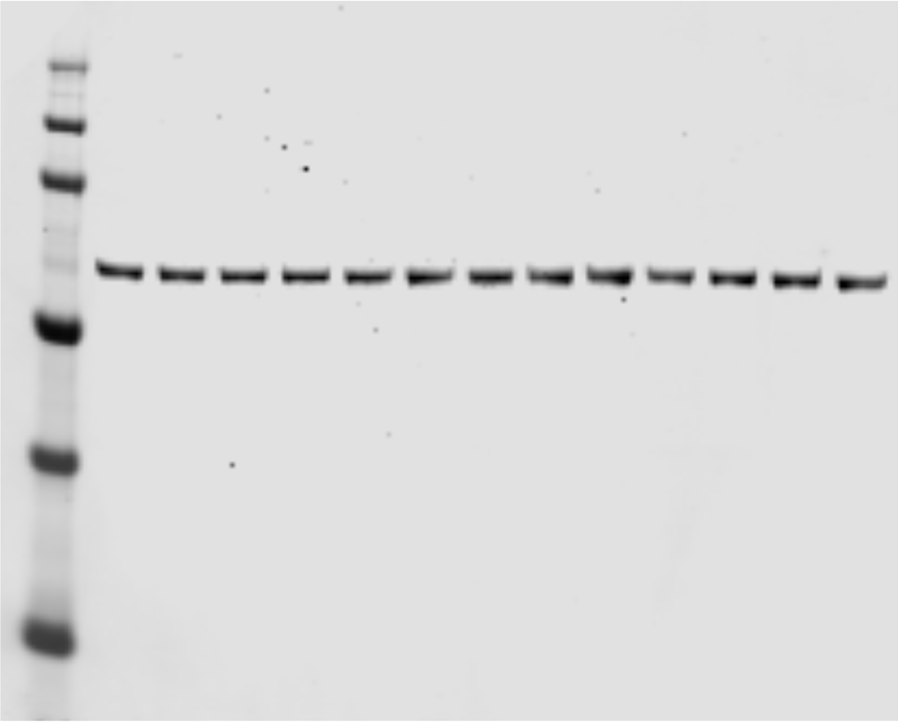

← SRC

replicate 2

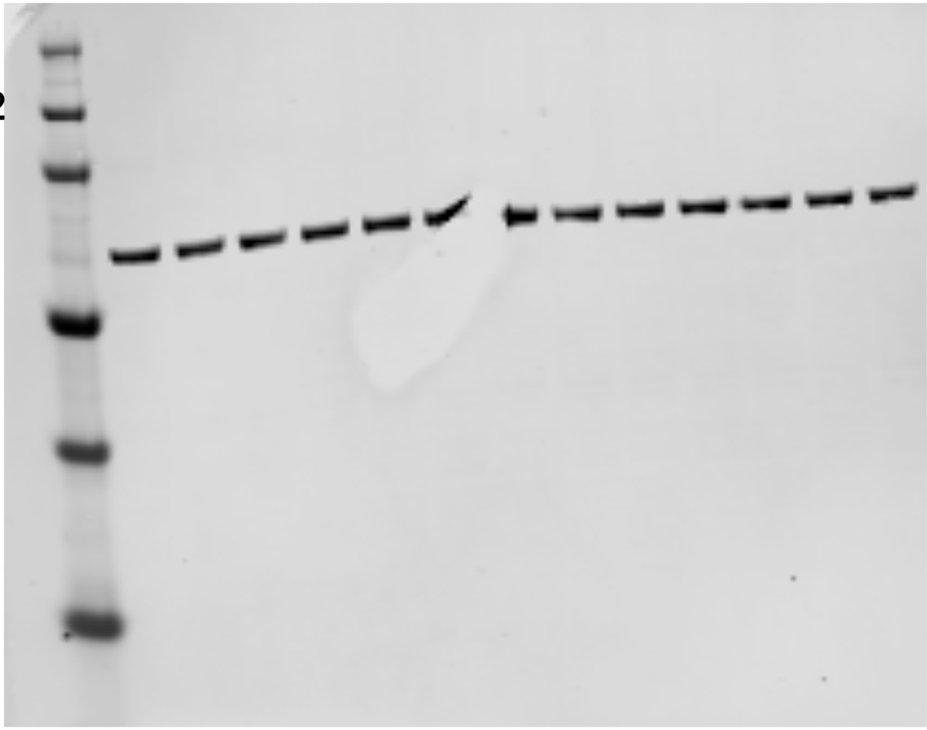

Supplement: Supplemental data [file jci-132-154604-s077.pdf]
